# A new mouse model to study restoration of interleukin-6 (IL-6) expression in a Cre-dependent manner: microglial IL-6 regulation of experimental autoimmune encephalomyelitis

**DOI:** 10.1186/s12974-020-01969-0

**Published:** 2020-10-15

**Authors:** Paula Sanchis, Olaya Fernández-Gayol, Gemma Comes, Kevin Aguilar, Anna Escrig, Mercedes Giralt, Richard D. Palmiter, Juan Hidalgo

**Affiliations:** 1 Institute of Neurosciences and Department of Cellular Biology, Physiology and Immunology, Animal Physiology Unit, Faculty of Biosciences, Universitat Autònoma de Barcelona, 08193 Barcelona, Spain; 2 Current affiliation: Department of Pediatrics, Division of Molecular Genetics, Columbia University Irving Medical Center, New York, 10032 USA; 3 Department of Biochemistry, Genome Sciences, and Howard Hughes Medical Institute, University of Washington, Seattle, WA 98195 USA

**Keywords:** DIO technology, Conditional reversible IL-6 knockout, Experimental autoimmune encephalomyelitis, Microglial IL-6

## Abstract

**Background:**

Interleukin-6 (IL-6) is a pleiotropic cytokine that controls numerous physiological processes both in basal and neuroinflammatory conditions, including the inflammatory response to experimental autoimmune encephalomyelitis (EAE). IL-6 is produced by multiple peripheral and central cells, and until now, the putative roles of IL-6 from different cell types have been evaluated through conditional cell-specific IL-6 knockout mice. Nevertheless, these mice probably undergo compensatory responses of IL-6 from other cells, which makes it difficult to assess the role of each source of IL-6.

**Methods:**

To give some insight into this problem, we have produced a novel mouse model: a conditional reversible IL-6 KO mouse (IL6-DIO-KO). By using double-inverted, open-reading-frame (DIO) technology, we created a mouse line with the loss of *Il6* expression in all cells that can be restored by the action of Cre recombinase. Since microglia are one of the most important sources and targets of IL-6 into the central nervous system, we have recovered microglial *Il6* expression in IL6-DIO-KO mice through breeding to *Cx3cr1*-CreER mice and subsequent injection of tamoxifen (TAM) when mice were 10–16 weeks old. Then, they were immunized with myelin oligodendrocyte glycoprotein 35-55 peptide (MOG_35-55_) 7 weeks after TAM treatment to induce EAE. Clinical symptoms and demyelination, CD3 infiltration, and gliosis in the spinal cord were evaluated.

**Results:**

IL6-DIO-KO mice were resistant to EAE, validating the new model. Restoration of microglial *Il6* was sufficient to develop a mild version of EAE-related clinical symptoms and neuropathology.

**Conclusions:**

IL6-DIO-KO mouse is an excellent model to understand in detail the role of specific cellular sources of IL-6 within a recovery-of-function paradigm in EAE.

## Introduction

Since their inception in the early 80s, genetically engineered mice have been a very powerful tool in biomedical research [1]. With time, the technology has been refined to allow for conditional knock-out of genes in specific cell populations [2] or in a timed manner [3]. However, the reversibility of these knockouts to allow for recovery of function experiments has been limited and somewhat flawed. Therefore, in this paper, we introduce a conditional (Cre-dependent) and reversible knockout for interleukin-6 (IL-6) using the double-inverted open reading frame (DIO) paradigm [4]. Our gene of choice for this proof-of-concept knockout was IL-6 since we had already successfully created a floxed mouse to achieve a conditional knockout [5]. The new mouse (IL6-DIO-KO) lacks IL-6 production in all cells, which can be turned back on by the action of Cre recombinase. In this paper, we demonstrate the knockout nature of this mouse in its initial form and test its applicability to specific research questions. We show the effect of selective recovery of IL-6 expression in microglia by crossing IL6-DIO-KO with Cx3cr1-CreER mice [6, 7] in the context of experimental autoimmune encephalomyelitis (EAE), the most popular mouse model of multiple sclerosis (MS).

IL-6 is a pleiotropic and multifunctional cytokine that mediates numerous physiological processes and is classically involved in acute and chronic inflammatory conditions [8]. Indeed, its overproduction has been reported in many pathological situations, including MS [9]. In the nervous parenchyma of patients with MS, IL-6 is predominantly linked to the active CNS plaques and located within resident glial cells concentrated in demyelinated areas [10]. IL-6 is also essential in the pathogenesis of EAE, as evidenced by IL-6 KO mice being resistant to it [11–14] and displaying altered markers of the disease such as reduced infiltration of T lymphocytes and monocytes into the central nervous system (CNS), reduced expression of adhesion molecules in endothelial cells, as well as defective differentiation of Th17 lymphocytes, which have been proposed as key factors leading to EAE resistance [11, 15]. In this context, and knowing that IL-6 regulates EAE in a source-specific way, given the varied results with conditional cell-specific IL-6 KO mice [16–19], we chose microglia as the cell population to test our reversible KO. Microglial cells have been described as one of the most important IL-6-producing brain cells in neuroinflammatory conditions and are clearly implicated in MS and EAE pathogenesis [20–23].

Our results show that this new IL6-DIO-KO mouse model is a useful tool to easily investigate the specific functions of IL-6 from each cellular source in EAE pathology. In particular, in the case of recovery of microglial IL-6, this was sufficient to initiate mild paralyzing symptoms related to EAE and slightly regulate the inflammatory cascade of EAE.

## Material and methods

### Animals

Mice were kept under constant temperature and a 12-h light:12-h dark with food and water available ad libitum. All experiments were approved by the Ethics Committee on Animal Experiments of the Universitat Autònoma de Barcelona and the Generalitat de Catalunya (Refs. 3782 and 9684, respectively).

#### Generation of IL6-DIO-KO mice

The conditional reversible IL-6 KO (IL6-DIO-KO) mice were developed using double-inverted open reading frame (DIO) technology [4]. Exon 2 of the *Il6* gene (*Il6*^*DIOex2*^) was inverted and flanked by two pairs of mutually incompatible loxP sites (lox2272 and loxP) in opposite orientations resulting in an inactive *Il6* allele. The action of Cre recombinase inverts exon 2 thereby restoring normal *Il6* expression.

**Fig. 1:**
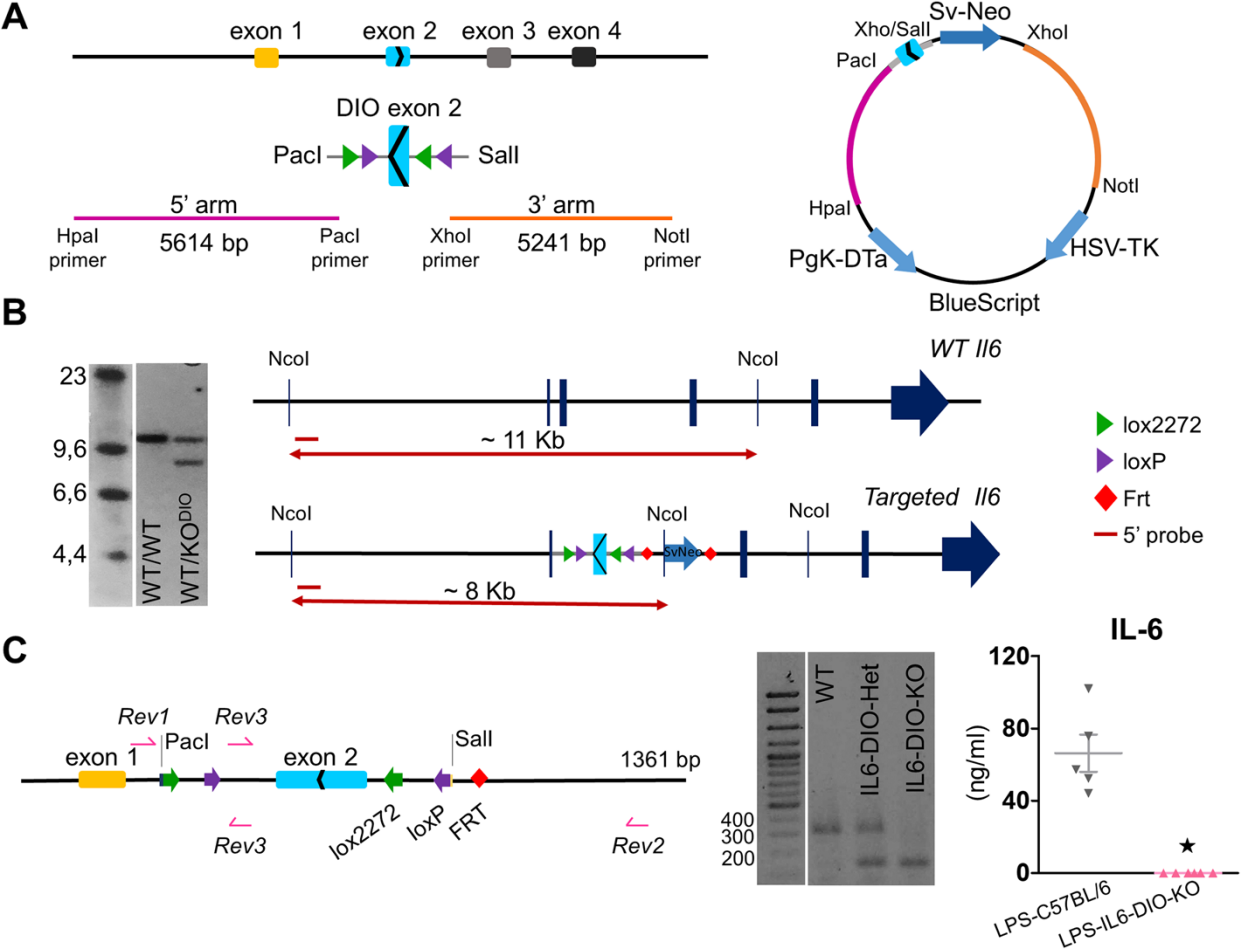
Generation of a conditional reversible KO mouse for IL-6. **a** Outline of the targeting strategy*. Left:* 5614 bp *HpaI-PacI* (pink; upstream) and a 5241 bp *XhoI-NotI* (orange; downstream) flanking *Il6*^*DIOex2*^ fragments. *Right:* the final product that consists of a *PacI-SalI* fragment with inverted exon 2 flanked by double lox sites, and three fragments containing *Pgk*-DT*a* and HSV-TK genes for negative selection and *frt*-flanked *SvNeo* gene for positive selection. **b** Southern blot for *Il6* gene of the positive ES clones using the probe (*left*) revealed a ~11 kb band on the wild type allele and a ~8 kb band on the targeted allele, as expected (*right*); those clones with two bands were selected. **c** *Left:* The *Il6* gene-modified sequence from IL6-DIO-KO mouse was sequenced by the Sanger method. The resulting sequence had 1361 bp and exon 1 (yellow), exon 2 (blue), lox2272 cassettes (green), loxP cassettes (purple), and FRT (red) were genetic structures observed in that sequence. *Middle*: PCR products for *Il6* gene of WT, IL6-DIO-Het, and IL6-DIO-KO mice yielded the expected bands. *Right*: IL-6 protein in serum was undetectable by ELISA in IL6-DIO-KO mice after LPS administration. All results are represented as mean ± SEM; ★*p* ≤ 0.05 vs. LPS-C57BL/6 mice

This mouse was generated as follows: a BAC clone from a C57Bl/6 J mouse containing the *Il6* sequence was obtained from Invitrogen (RP23-121 M2). A 5614 bp fragment with engineered *HpaI* and *PacI* sites upstream of exon 2 and a 5241 bp fragment with engineered *XhoI* and *NotI* sites downstream of exon 2 of the *Il6* gene were synthesized by PCR (using Q5 polymerase, NE Biolabs) (Fig. 1a, left). Exon 2 flanked by double lox sites with *PacI* and *SalI* sites at the ends was synthesized by Genscript and inserted into a targeting vector in the reverse orientation. The three fragments were sequentially cloned into a targeting vector containing *Pgk-DTa* and *HSV-TK* genes for negative selection and *frt*-flanked *SvNeoR* for positive selection. The final construct was purified from a CsCl gradient, linearized with *AscI* and electroporated into G4 hybrid (C57Bl/6 × Sv129) embryonic stem (ES) cells (Fig. 1a, right). Correctly targeted clones were identified by Southern blot; approximately 5 μg of DNA from ES cells were digested with *NcoI*, electrophoresed on a 0.7% agarose gel and transferred to a nylon membrane. Next, the membrane was hybridized to a unique probe of 621 bp located outside the 5’ arm (synthesized using the primers *Fw*: 5’-CAGCATCTCATCTGAGTTCCG-3’ and *Rv*: 5’-CTCACTGTTCACAAAGCACAGG-3’) (Additional file 1) that would give a ~11 kb band for the wild-type allele and a ~8 kb band for the targeted allele (Fig. 1b). Clones with the two bands were reassessed using Neo as a probe. Four positive clones (out of 81 analyzed) were correctly targeted and had a single NeoR gene insert. Three of those were injected into C57Bl/6 blastocysts, which were implanted in receptive females. Those mice with a high percentage of chimerism were then crossed with *Gt(ROSA)26Sor-FLP* recombinase to remove the Sv-NeoR gene [24]. Mice heterozygous for the *Il6*^DIOex2^ gene were backcrossed >10 times to C57BL/6OlaHsd mice, and non**-**littermate heterozygous mice for *Il6*^DIOex2^ gene from the tenth crossing were crossed to each other to obtain three possible genotypes: *Il6*^DIOex2/DIOex2^ (IL6-DIO-KO mice), *Il6*^WT/WT^ (WT mice) and *Il6*^DIOex2/WT^ (IL6-DIO-Het mice, with one functional *Il6* allele).

#### Reactivation of microglia-derived *Il6* expression

IL6-DIO-KO mice were crossed with heterozygous Cx3cr1-CreER mice, produced by Drs. Jung and Prinz and obtained from the European Mouse Mutant Archive (EMMA) repository, where were backcrossed with C57BL/6 mice more than 10 generations [7]. Then, the offspring Cre-positive animals from the first crossing were selected and crossed again with homozygous IL6-DIO-KO mice to obtain mice homozygous for IL6-DIO-KO and carrying Cx3cr1-CreER allele; these mice are designated IL6-DIO-ON^Cx3cr1^ and should have *Il6* expression only where Cx3cr1 gene is expressed, including microglia.

### Genotyping

Mice were genotyped by PCR analysis of tail and liver DNA, extracted previously by boiling in 100 μl of 50 mM sodium hydroxide for 7 min, using three primers (*Rev1*: 5’-GAGACTGTGAGAGAGGAGTGTG-3’; *Rev2*: 5’-CATCTTATCTGGGCTGACCCTAG-3’; *Rev3*: 5’-TCTCTGCTGGGATCTAGGGCC-3’) and the following reaction conditions: 95 °C 2 min, followed by 30 cycles of 95 °C 30 s, 52 °C 30 s, and 72 °C 30 s, and 72 °C 5 min. Finally, *Cre* primers and PCR conditions were adapted from Sanz et al. [25].

### Sanger DNA sequencing

The modified fragment of *Il6* gene from IL6-DIO-KO mouse was sequenced by the Sanger method. Two PCR products were amplified separately using two pairs of primers. The first pair of primers amplified from the middle of the first exon to the end of the third intron (*1F*: 5’-CCCACCAAGAACGATAGTCA-3’ and *ΔR*: 5’-ATGCCCAGCCTAATCTAGGT-3’ [5] while the second pair amplified from the first intron to the end of the second intron (*fw*: 5’-CGATGCTAAACGACGTCACA-3’and *Rev3* primer). Then, the PCR fragments were purified by ExoSAP-IT Express PCR Cleanup Reagent (ThermoFisher, 75001). The enzymatic cleanup method and sequencing were performed by Servei de Genòmica i Bioinformàtica (IBB-UAB). The data were imported firstly into Snapgene Viewer 4.3.4. Software (from Insightful Science; available at snapgene.com), in which both sequences were overlapped creating a unique sequence with 1361 bp.

### Tamoxifen treatment

Cx3cr1-CreER mouse is a tamoxifen (TAM)-inducible model [7], then the IL6-DIO-ON^Cx3cr1^ mice and their littermates were injected for five or eleven consecutive days with TAM. Their littermates were also administered with TAM to control the effects of TAM in the analyzed parameters. TAM (Sigma 5648) was dissolved in ethanol at a concentration of 10 mg/100 μl by shaking and heating at 37 °C [26]; next, the solution was diluted with sunflower oil to 10 mg/ml and one daily dose of 100 μl (1 mg) was injected intraperitoneally to each mouse.

### Lipopolysaccharide injection

Mice were injected intraperitoneally with 0.5 mg/Kg lipopolysaccharide (LPS O55:B5, Sigma L2880) and blood was drawn from the tail 90 min later; it was centrifuged at 9.500x*g* for 10 min at 4 °C and serum was stored at −80 °C before testing.

### Microglia isolation

IL6-DIO-KO and IL6-DIO-ON^Cx3cr1^ mice were euthanized 1 month after 11 days of TAM treatment and brains (excluding cerebellum) were quickly dissected and chopped in small pieces. Then, the tissue was dissociated both enzymatically and mechanically using the adult brain dissociation kit (Miltenyi 130-107-677) and placing it on a gentleMACS Octo Dissociator for 30 min at 37 °C. After dissociation, debris and erythrocytes were removed and microglia were isolated using magnetic microbeads against Cluster of differentiation molecule 11b (CD11b; Miltenyi 130-093-634) following the manufacturer’s instructions. The purity of microglia in CD11b+ cell population was previously checked in [26]. The CD11b-negative flow-through was named “remaining sample”. DNA from cells was extracted as described above.

### Induction of EAE and clinical evaluation

The induction of EAE was carried out using adult mice. All animals were immunized under isoflurane anesthesia (oxygen flowmeter to 0.8 L/min and isoflurane vaporizer to 4 and 1.5% during induction and maintenance, respectively) as described [19]. On day 0, mice were injected subcutaneously into the hind flanks with an emulsion of 100 μL MOG_35–55_ (3 mg/ml; sequence: MEVGWYRSPFSRVVHLYRNGK-carboxyl. Purity: >98%. Peptide synthetized upon request in the Peptide Synthesis Facility at the Universitat Pompeu Fabra) and 100 μL of complete Freund’s adjuvant (CFA; Sigma-Aldrich F5881) supplemented with 3 mg/ml *Mycobacterium tuberculosis* H37RA (BD Difco 231141). Control mice were immunized with an emulsion containing bovine serum albumin (BSA; 3 mg/ml; Sigma A9647) instead MOG_35–55_ peptide. All mice received an intraperitoneal injection of 500 ng *Bordetella pertussis* toxin (Native Antigen Company PT-TNL-50) on day 0 following MOG_35-55_/BSA injection and on day 2 post-immunization. *Bordetella pertussis* toxin is widely used to facilitate the induction of EAE since it destabilizes the blood-brain barrier and facilitates the migration of T cells into the CNS.

After immunization, body weight and clinical score were monitored daily. The progression of the disease was assessed in each animal with a numerical scale following these criteria: 0 = no clinical signs, 0.25 = slight loss of tail tonus, 0.5 = partial loss of tail tonus, 1 = paralyzed tail, 2 = moderate hind limb paraparesis, 2.5 = severe hind limb paraparesis, 3 = partial hind limb paralysis, 3.5 = hind limb paralysis, 4 = tetraplegia, and 5 = death. When mice lost more than 10% weight, they were injected intraperitoneally with 200 μl of saline buffer supplemented with 3.6% glucose. We applied the following endpoint criteria: (i) self-mutilation; (ii) weight loss greater than 25%; or (iii) tetraplegia for more than 2 days. The time to disease onset and time to peak disease (days to peak score) were defined by a clinical score ≥1. The peak score (highest score) and median score were also calculated individually.

Mice were euthanized by decapitation and their spine was cut between the ninth and tenth thoracic vertebrae. The upper part was processed for histological analysis (see “Histology” section) and the lower part was cut open to extract the spinal cord (segments T12-Co3), which was snap-frozen in liquid nitrogen and stored at −80 °C for molecular analysis of the inflammatory response. Blood was collected from the trunk and the serum was obtained as mentioned before and stored at −80 °C.

### IL-6 enzyme-linked immunosorbent assays (ELISA)

IL-6 levels in serum were measured using IL-6 ELISA following manufacturer’s instructions (Bionova 860020192).

### Real-time polymerase chain reaction (qPCR)

RNA was extracted using Promega Maxwell RSC simplyRNA kit following the manufacturer’s instructions. Then, 2 μg of RNA of each sample were treated with an extra DNase I step (Qiagen). After removing DNA from the RNA samples, a two-step qPCR protocol was performed using iScript cDNA synthesis kit (BioRad) and iTaq Universal SYBR Green Supermix (Bio-Rad Laboratories 1725124). Fold change expression was calculated with an adaptation of delta-delta-Ct method using Glyceraldehyde 3-phosphate dehydrogenase (*Gapdh*) as the reference gene and the EAE IL6-DIO-Het group as calibrator.

### Histology

The upper part of the vertebral column with the spinal cord from sham and MOG_35-55_-immunized mice was fixed with 4% PFA for 24 h. Afterwards, they were carefully dissected, being the spinal cord post-fixed for another 24 h in 4% PFA, subsequently embedded in paraffin and cut sagittally into 8-μm-thick sections. Two non-consecutive slices from each animal were used for each histological analysis. For myelin evaluation, samples were stained with 0.1% Luxol Fast Blue (LFB) overnight at 56 °C. The following day, the tissue was differentiated with 0.05% lithium carbonate (Fluka, 62 K70) and 70% ethanol, and counterstained with hematoxylin solution (Sigma MHS16). For IHC analysis, microglia/macrophages, astrocytes and lymphocytes cluster of differentiation 3+ (CD3+) were identified by antibodies against ionized calcium-binding adaptor molecule-1 (IBA-1; Wako 019-19741, 1/1500), Glial fibrillary acidic protein (GFAP; Dako Z0334, 1/900), and CD3+ (Dako A0452, 1/100), respectively.

Antigen retrieval was necessary in IBA-1 and CD3 IHC. For the former, samples were treated with 10 mM sodium citrate 0.05% tween for 20 min at 96 °C, whereas for the latter, they were incubated with 1 g/l protease type XIV (Sigma, P5147) for 8 min at 37 °C. Then, samples were incubated with endogenous peroxidase blocking buffer (70% methanol and 3% hydrogen peroxide (H_2_O_2_)) for 15 min and then blocked in 1% BSA in 0.05 M Tris-buffered saline and 0.5% triton-X100 for 1 h. The incubation with primary antibodies was overnight at 4 °C. The following day, tissues were incubated with a biotin-conjugated secondary antibody Atom BA-1000 (1/300) for 1 h and then, with horseradish peroxidase-coupled streptavidin (Vector SA-5004, 1/500-600). Immunoreactivity was visualized by adding 50 mg/ml 3,3-diaminobenzidine-tetrahydrochloride and 0.033% H_2_O_2_ for 1–5 min, depending on the primary antibody. Samples for CD3 were counterstained with hematoxylin solution (Sigma MHS16-500 ml).

Serial images of the tissues from duplicates of each animal were taken at ×10 using a Nikon Eclipse 90i microscope and Nikon Act-1 software. Quantification was performed with ImageJ software [27], using a color deconvolution plugin [28] in CD3 IHC counterstained with hematoxylin and LFB/hematoxylin staining to separate both colors and quantify only CD3+ cells and myelin, respectively. In LFB staining, the percentage of demyelination was evaluated in the white matter (WM). For CD3 IHC, we quantified the integrated density (sum of all the pixel intensities in the region of interest) of CD3 per infiltrate. In IBA-1 immunostaining, manual count of IBA-1+ cells in the white and gray (GM) matter of the spinal cord was carried out in the first EAE experiment whereas the quantification of the integrated density of white and gray matter, as well as the manual count of IBA-1+ cells in the white matter, were performed in the second EAE experiment. In addition, we characterized IBA-1+ cells in terms of their morphology. IBA-1+ cells with long and thin processes (ramified morphology) were classified as resting IBA-1+ cells whereas those with an enlarged body and small process were considered reactive IBA-1+ cells. IBA-1+ cells with a round body were classified as fully activated cells. In GFAP immunostaining, manual count of GFAP + cells were performed in the GM in the first experiment; however, this was not possible in the white matter given its background was too high. The quantification of the integrated density of white and gray matter, as well as manual count of GFAP + cells in the GM gray matter, were carried out in the second EAE experiment. Furthermore, those cells with thin and reduced processes (ramified morphology) were classified as resting GFAP+ cells whereas those with thicker hypertrophic processes (star-shaped morphology) were recorded as reactive GFAP+ cells. All results were normalized to the measured area.

### Statistics

Data is shown as mean ± SEM, plus individual data points (except for clinical score), except for EAE summary tables, where median and interquartile range (IQR) are used. Statistical calculations were carried out using the Statistical Package for Social Sciences software (SPSS, version 19) unless otherwise stated. The clinical score was analyzed with a generalized linear model (GzLM) using the area under the curve (AUC), which had been previously calculated using GraphPad Prism. Body weight gain and incidence were analyzed by generalized estimated equations (GEE) and Fisher’s exact test, respectively. The day of onset (with score ≥1) and time to peak (with score ≥1) were examined using Kaplan–Meier survival analysis plus log-rang test, and the post-hoc pairwise comparisons were performed using the Holm-Bonferroni correction (R packages “survival” and “survminer”; RStudio (version 1.2.5033, with R version 3.6.3)). Peak score and median score were analyzed by Mann–Whitney U test to compare two groups or Kruskal–Wallis rank test and Dunn’s test (post hoc) to compare three groups. Histological and gene expression results were analyzed by GzLM using genotype as the main factor and a post hoc sequential Bonferroni test was carried out when the main factor was significant. In the case of comparisons between two groups, the Student *t* test was used. Outlier detection was carried out using the interquartile range (IQR) rule computed from Tukey’s hinges, and values more than 3 IQR’s from the end of the box were considered potential outliers and removed from the statistical analysis of histological and gene expression results. Heatmaps to show IL6 levels (or gene expression) alongside AUC were created with the R package “pheatmap” with no clustering and color breaks set with quantiles. Statistical significance was set at *p* ≤ 0.05 for all analyses.

## Results

### Generation and characterization of IL6-DIO-KO mice

We created a new murine model with an inactive *Il6* allele (see “Generation of IL6-DIO-KO mice” section) that can be specifically reactivated by the action of Cre recombinase (Fig. 1), using a strategy based on DIO technology [4, 29–31]. In the absence of Cre recombinase, homozygous mice are null for *Il6* expression (exon 2 is inverted) and designated as IL6-DIO-KO. The action of Cre recombinase flips exon 2 so that it can be spliced normally thereby restoring *Il6* expression; these mice are referred to as IL6-DIO-ON with a superscript indicating what Cre-driver line was used.

We sequenced the modified *Il6*^DIOex2^ gene of IL6-DIO-KO mice by the Sanger method. By using two pairs of primers, we obtained two sequences that were overlapped to create a single genetic map of 1361 bp (Fig. 1c, left and Additional file 2). As expected, 2 pairs of lox cassettes are flanking exon 2 of the *Il6* gene on each side (LoxP and Lox2272), two heterologous loxP sites that cannot recombine with each other. In addition, one residual Frt (FLPase recognition target [32];) site is also present. The result of the sequencing showed the inverted exon 2 resulting in an exon that cannot be spliced due to lack of splice acceptor and donor sites.

Three primers were designed for the routine genotyping of the normal and inverted exon 2 (see “Genotyping” section). By using *Rev3* and *Rev2*, a product of 358 bp was amplified for the WT allele of WT and IL6-DIO-Het mice, whereas using *Rev3* and *Rev1* primers, a 193 bp band was obtained for the KO allele of IL6-DIO-Het and IL6-DIO-KO mice (Fig. 1c, middle). Analysis of IL-6 production in IL6-DIO-KO and non-littermate control mice was carried out in serum collected 90 min after a 0.5 mg/kg LPS injection. IL-6 protein levels were dramatically increased by LPS in C57BL/6OlaHsd mice, but they remained absent in LPS-IL6-DIO-KO mice (Fig. 1c, right).

**Fig. 2:**
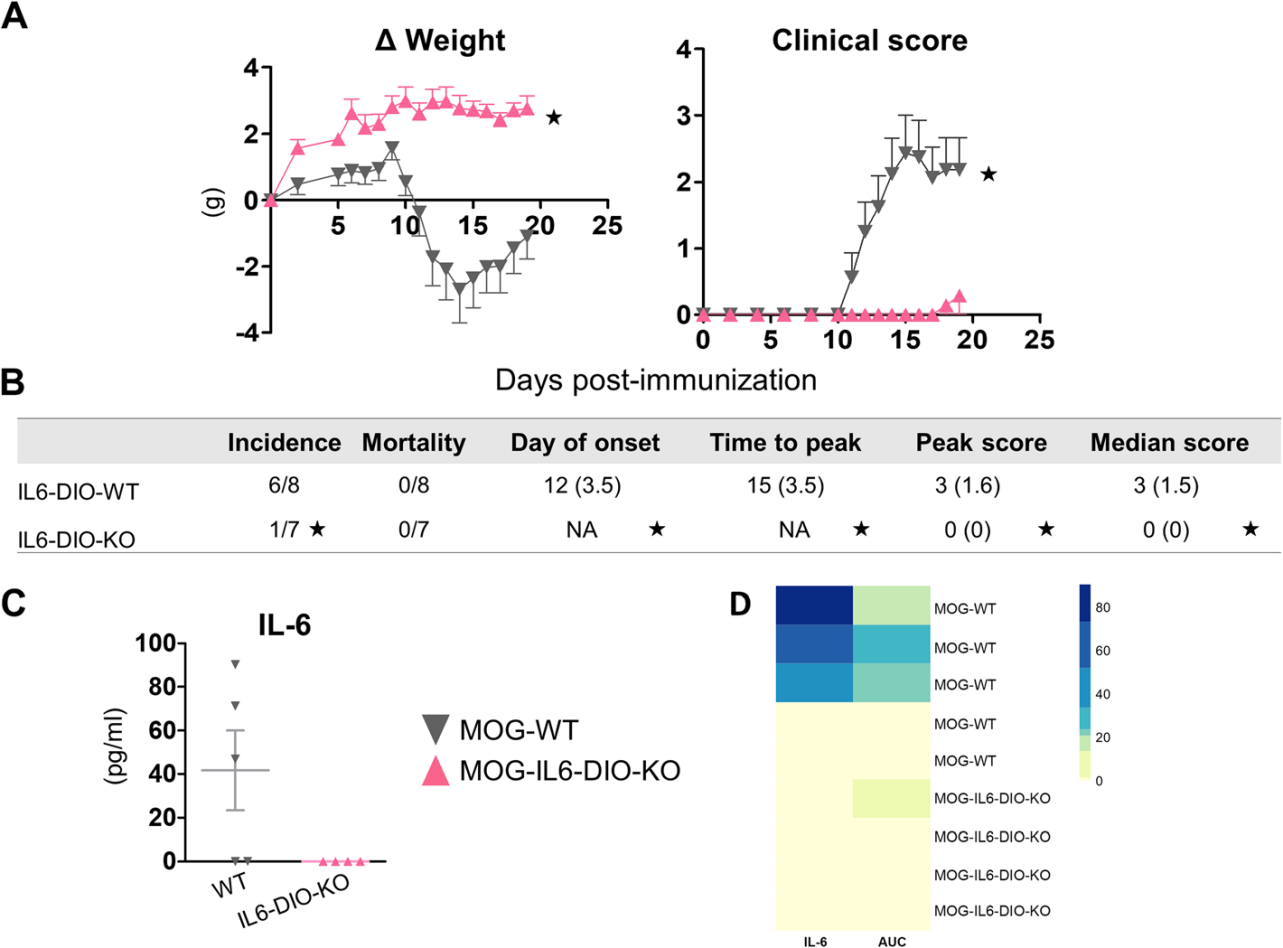
IL6-DIO-KO mice are resistant to EAE. **a** *Left*: Weight loss of WT and IL6-DIO-KO mice after being immunized with MOG_35–55_ peptide. *Right*: Clinical evaluation of MOG_35-55_-immunized WT and IL6-DIO-KO mice. IL6-DIO-KO mice did not develop EAE-related symptoms. **b** Clinical evaluation of EAE. A significant effect on the disease onset, time to peak disease, peak score, and median score between WT and IL6-DIO-KO mice was observed. The incidence, disease onset, and time to peak were calculated using a clinical score ≥1. **c** Serum IL-6 protein levels were not detected in IL6-DIO-KO mice at 19 dpi. **d** Heatmap of IL-6 ELISA levels and the respective AUC for each animal in the experiment. Mice with higher disease scores also showed higher IL-6 levels. It is also clear that the two WT mice that did not develop clinical symptoms also did not have increased IL-6 levels. All results are represented as mean ± SEM; ★*p* ≤ 0.05 vs. MOG-WT mice

IL6-DIO-KO mice and littermate WT controls were immunized with the MOG_33-55_ peptide, and the clinical course and body weight were monitored for 19 days post-immunization (dpi). MOG-WT mice showed the prototypical course of ascending paralysis starting at 12 dpi (IQR 3.5) and peaking approximately at 15 dpi (IQR 3.5) with a clinical score of 3 (IQR 1.6) (Fig. 2a, *right* and 2b). They also showed a prominent body weight loss in parallel with the appearance of clinical signs (Fig. 2a, *left*). MOG-IL6-DIO-KO mice were resistant to EAE since only 14% showed minor paralyzing symptoms. The day of disease onset, peak score, time to peak, and median score were all statistically significant between genotypes (Fig. 2b and Additional file 3). As expected, IL-6 protein was undetectable in serum samples from MOG-IL6-DIO-KO mice, but present in their controls (Fig. 2c). Furthermore, a heatmap of IL-6 levels and area under the curve was performed, reinforcing that the idea that mice with more severe EAE-related symptoms also show more serum IL-6 levels (Fig. 2d).

**Fig. 3:**
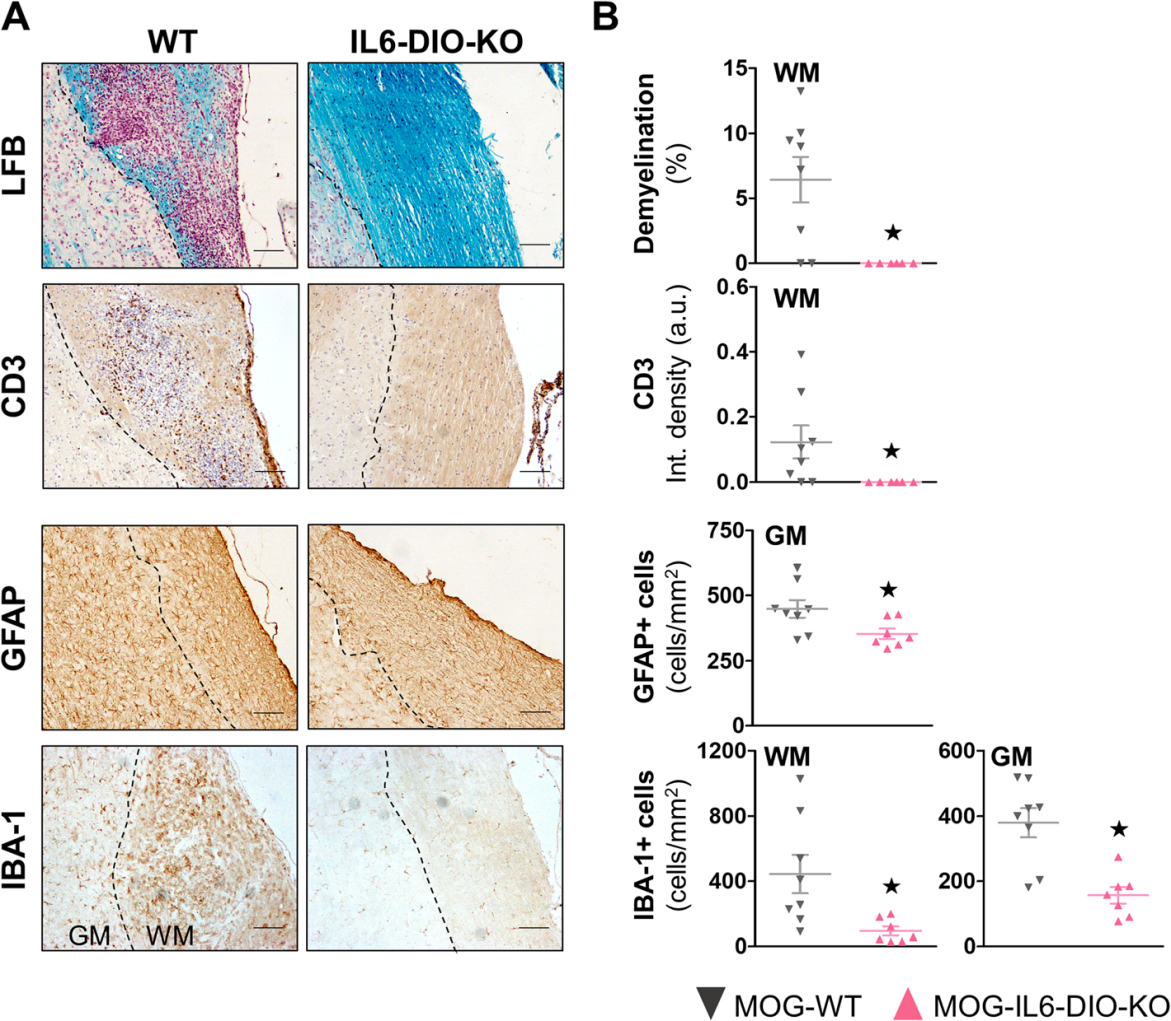
MOG_35-55_-immunized IL6-DIO-KO mice showed an absence of demyelination and CD3 infiltrates, and limited gliosis. **a** Representative images of LFB staining and CD3 infiltrates (*top*), astrogliosis and microgliosis (*bottom*) of the spinal cord from MOG-WT and MOG-IL6-DIO-KO mice. The contrast of the representative images was enhanced. The lack of IL-6 prevented the infiltration of inflammatory cells. The discontinuous line delimits white matter (WM) from gray matter (GM). Scale bar: 100 μm. **b** *From top to bottom:* Quantification of demyelination by LFB staining and CD3 immunostaining levels (*top*) and the total number of GFAP+ and IBA-1+ cells (*bottom*) in the spinal cord from MOG-WT and MOG-IL6-DIO-KO mice. All results are represented as mean ± SEM; ★*p* ≤ 0.05 vs. MOG-WT mice

The upper part of the spinal cord (segments C1–T11) from MOG-WT mice, but not those from MOG-IL6-DIO-KO mice, showed clear signs of focal demyelination at 19 dpi, in agreement with clinical signs. Infiltration of CD3+ T cells was also prominent in the white matter of MOG-WT mice whereas it was practically absent in MOG-IL6-DIO-KO mice (Fig. 3a and b, top). We also counted both the number of GFAP+ and IBA-1+ cells along the spinal cord since astrocytes (GFAP+ cells) and microglia /macrophages (IBA-1+ cells) since they play an important role in EAE pathogenesis mediating the inflammatory response. A more prominent accumulation of GFAP+ cells in the gray matter and IBA-1+ cells both in the white and gray matter were seen in spinal cords from MOG-WT mice, and to a lesser extent in those from MOG-IL6-DIO-KO mice (Fig. 3a and b, bottom).

### IL6-DIO-ON^Cx3cr1^ mice showed genetic rearrangement of *Il6* specifically in microglia 1 month after TAM injections

**Fig. 4:**
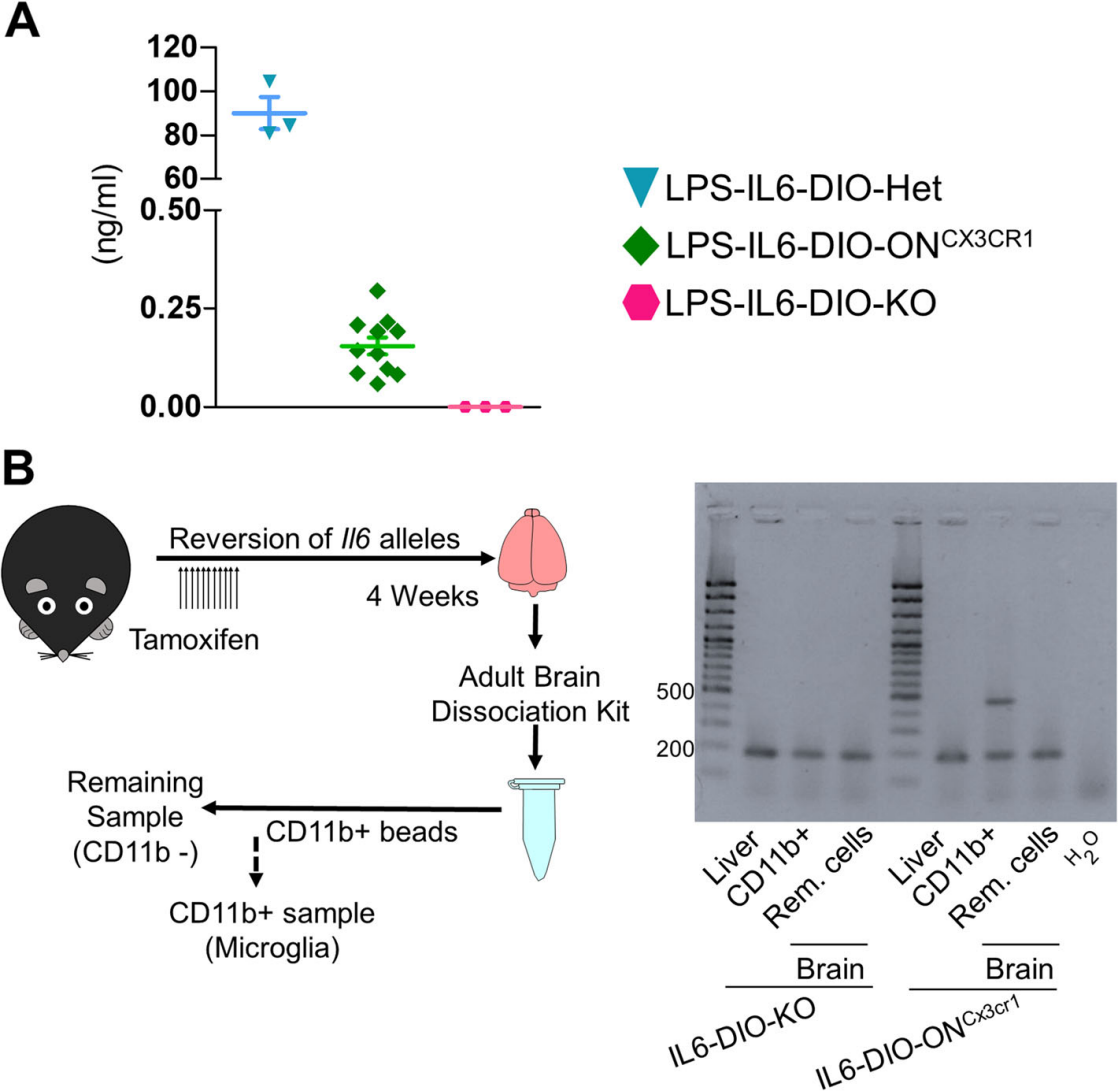
Reactivation of microglia-derived *Il6* expression in IL6-DIO-ON^Cx3cr1^ mice. **a** Serum IL-6 protein levels of LPS-injected IL6-DIO-Het, IL6-DIO-ON^Cx3cr1^ and IL6-DIO-KO mice 1 day following 11 days of TAM treatment. **b** *Left*: IL6-DIO-ON^Cx3cr1^ and IL6-DIO-KO mice were euthanized 1 month after TAM treatment and the brains were homogenized and incubated with CD11b+ antibody. Besides, the positive cell population was selected using columns with ferromagnetic spheres. *Right*: The reversion band (463 bp) was only seen in the brain CD11b+ fraction of IL6-DIO-ON^Cx3cr1^ mice. All results are represented as mean ± SEM

We evaluated the ability to reactivate *Il6* expression selectively in Cx3cr1-expressing cells and defined the duration of TAM exposure for optimal inversion of exon 2 specifically in Cx3cr1-expressing cells. First, we treated 13- to 17-week-old mice with TAM for 5 days (1 mg/day) and 2 weeks afterwards injected them with LPS. We detected IL-6 in the serum of none of LPS-IL6-DIO-KO mice, all of LPS-IL6-DIO-Hz animals, but only in two out of six LPS-IL6-DIO-ON^Cx3cr1^ animals, showing that recombination was insufficient (data not shown). Since exon 2 inversion did not work with 5 days of TAM treatment, 10- to 16-week-old mice were treated with TAM for 11 days, and with LPS 1 day after the TAM treatment ended. Serum IL-6 now increased modestly in all LPS-injected IL6-DIO-ON^Cx3cr1^ mice while all LPS-treated IL6-DIO-KO controls remained undetectable. LPS-treated IL6-DIO-Het mice produced high levels of IL-6, as expected (Fig. 4a). This protocol was therefore established for subsequent use.

To test the specificity of recombination in microglial cells, we reactivated *Il6* expression in Cx3cr1-expressing cells by treating with TAM for 11 days and, 4 weeks later, extracted DNA from the liver (as a control) and the brain, separating its cells in microglia (CD11b+ sample) and the remainder (CD11b- sample) (Fig. 4b, left). This control is necessary because in *Cx3Cr1*-CreER mice, CreER is driven by the *Cx3cr1* promoter, which is expressed in all mononuclear phagocytic cells, which include macrophages [7]. Also, DNA inversion should be present longer in microglia than in Cx3cr1-expressing peripheral cells given microglia’s longevity. Therefore, we showed a partial reversion of *Il6* in IL6-DIO-ON^Cx3cr1^ mice by PCR detection of the reversed band (463 bp) in microglia (CD11b+ sample) but not in a liver sample (where presumably there are circulating Kupffer cells and/or mononuclear cells). As expected, the inverted exon 2 band was observed in both tissues from IL6-DIO-KO mice (Fig. 4b, right).

### Reactivation of *Il6* expression in microglia partially rescues EAE pathology

IL6-DIO-Het, IL6-DIO-ON^Cx3cr1^, and IL6-DIO-KO females were used to physiologically confirm *Il6* reversion in Cx3cr1-expressing cells. These mice were immunized with MOG_35-55_ peptide 7 weeks after being injected with a total of 11 mg of TAM. A group of BSA-injected female and male IL6-DIO-Het mice (but not injected previously with TAM either LPS) was also included as a control for illustrating the neuropathology of EAE.

**Fig. 5:**
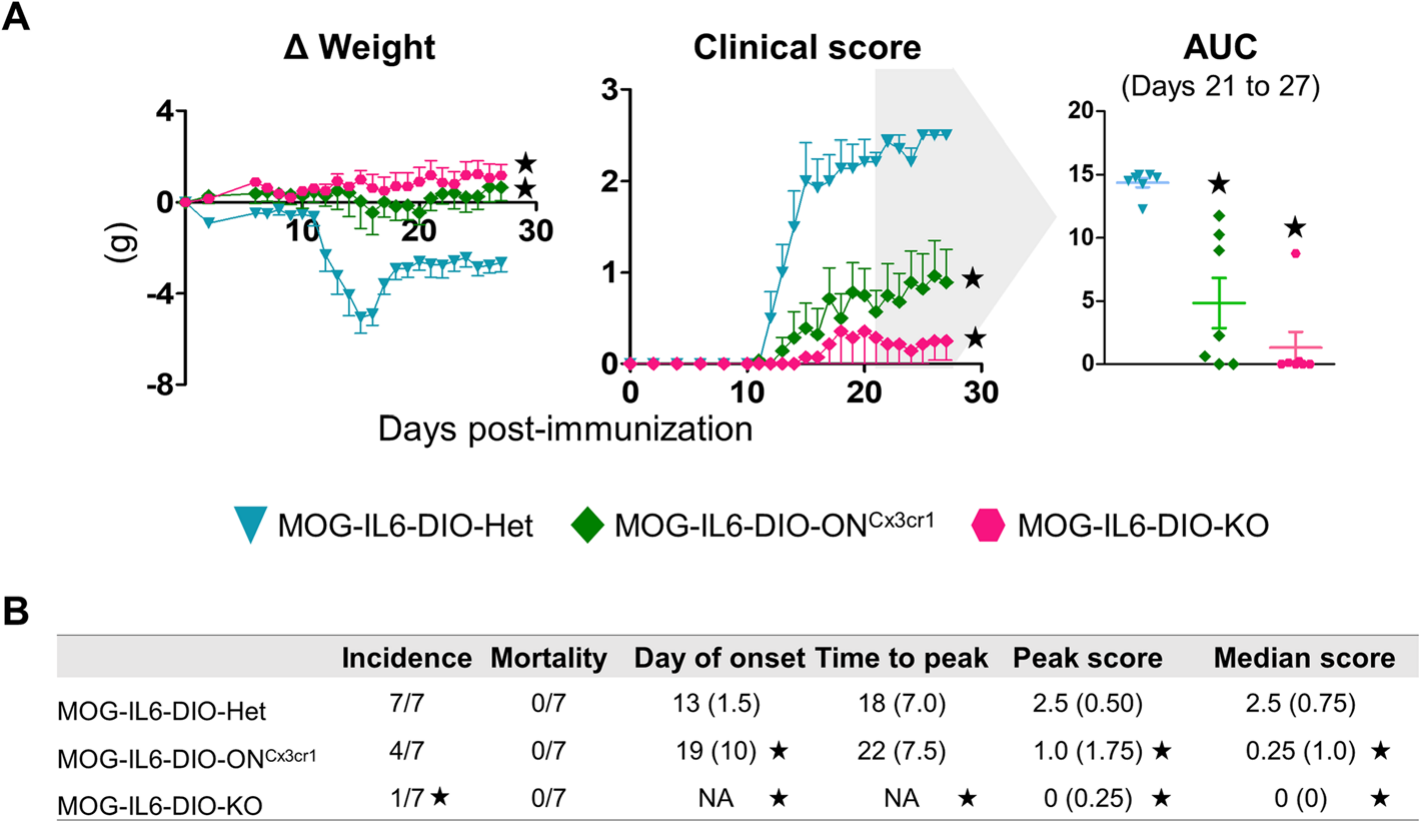
IL6-DIO-ON^Cx3cr1^ showed mild EAE-related symptoms. **a** Clinical course of MOG-IL6-DIO-Het, MOG-IL6-DIO-ON^Cx3cr1^ and MOG-IL6-DIO-KO mice. Body weight (*left*) and clinical signs (*middle*) changes were observed in IL6-DIO-Het and IL6-DIO-ON^Cx3cr1^ mice whereas IL6-DIO-KO mice were almost completely resistant to EAE. Area under the curve (AUC; *right*) was calculated from EAE clinical course for each mouse between days 21 and 27. **b** Clinical evaluation of EAE. IL6-DIO-ON^Cx3cr1^ and IL6-DIO-KO mice were significantly less affected than IL6-DIO-Het mice. The incidence, disease onset, and time to peak were calculated using a clinical score ≥1. Results in (**a)** are represented as mean ± SEM, while (**b**) shows median and IQR (except for incidence and mortality). NA (not applicable) shows the impossibility to calculate the median (given the criterion for disease). ★*p* ≤ 0.05 vs. MOG-IL6-DIO-Het mice

Following MOG_35-55_ immunization, MOG-IL6-DIO-Het mice showed the typical ascending paralysis course of EAE, starting at 13 dpi (IQR 1.5) and peaking at 18 dpi (IQR 7) with a clinical score of 2.5 (IQR 0.5) (Fig. 5a, middle and Fig. 5b). Moreover, a prominent body weight loss occurred in these mice in parallel with the clinical signs (Fig. 5a, left). Consistent with our initial results, MOG-IL6-DIO-KO mice were almost completely resistant to EAE (Fig. 5a, b). In contrast, mice with the recovery of *Il6* only in microglia (MOG-IL6-DIO-ON^Cx3cr1^) showed a delayed progression of the clinical course, with paralyzed tail at 19 dpi (IQR 10) and peaking at 22 dpi (IQR 7.5), with a peak score of 1 (IQR 1.75) (Fig. 5a and b). As shown in Fig. 5b, the incidence of disease symptoms (clinical score ≥1) was 100% in MOG-IL6-DIO-Het and 57% in MOG-IL6-DIO-ON^Cx3cr1^ mice. No mice died during the experiment (27 days) (Fig. 5b).

Significant differences regarding EAE symptomatology were observed between MOG-IL6-DIO-Het and MOG-IL6-DIO-KO mice, and between MOG-IL6-DIO-Het and MOG-IL6-DIO-ON^Cx3cr1^ mice. Furthermore, the severity of the disease was also analyzed by the evaluation of the AUC for each mouse from 21 to 27 dpi. We observed differences between MOG-IL6-DIO-Het and MOG-IL6-DIO-ON^Cx3cr1^ mice (*p* < 0.001), and MOG-IL6-DIO-Het and MOG-IL6-DIO-KO mice (*p* < 0.001). Regarding the day of disease onset, peak score, and median score, MOG-IL6-DIO-Het mice were significantly different from both MOG-IL6-DIO-ON^Cx3cr1^ and MOG-IL6-DIO-KO mice (Fig. 5b and Additional file 4). Immunization with BSA did not cause any paralyzing symptoms in IL6-DIO-Het mice. Furthermore, they maintained body weight for all days analyzed, as expected (data not shown).

**Fig. 6:**
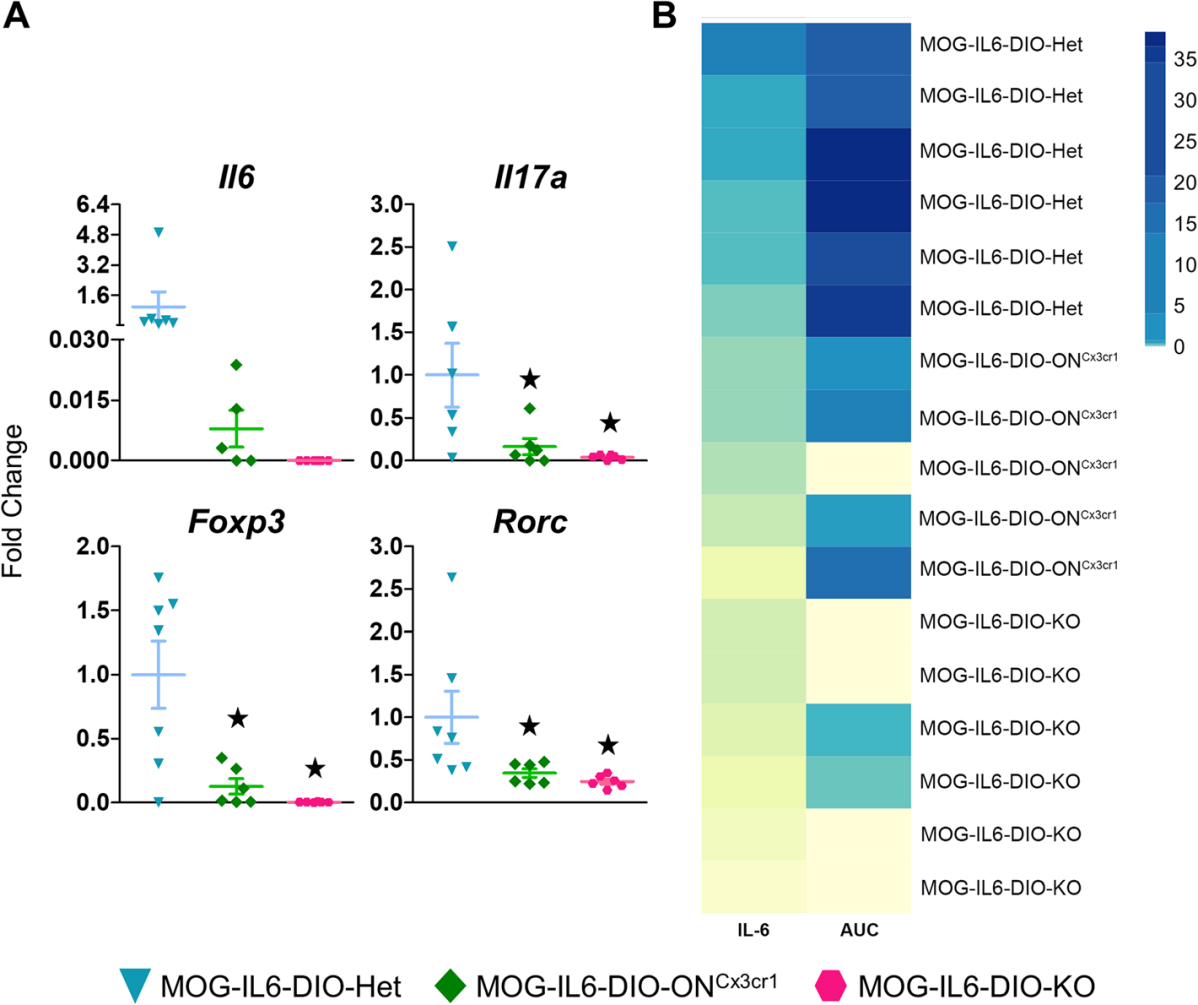
Inflammation-related genes are down-regulated accordingly to disease severity**.** The changes of inflammation-related genes expression were studied using the upper part of the spinal cord (segments C1–T11) of three groups of mice euthanized at 27 dpi. **a** Gene expression of *Il6*, *Il17a*, *Foxp3,* and *Rorc* was lower in MOG-IL6-DIO-ON^Cx3cr1^ and MOG-IL6-DIO-KO mice than in MOG-IL6-DIO-Het control mice. **b** Heatmap of *Il6* expression and AUC of disease score. *Il6* was only increased in mice with higher AUC. Results in (**a**) are represented as mean ± SEM; ★*p* ≤ 0.05 vs. MOG-IL6-DIO-Het mice

*Il6* mRNA levels were higher in the lower part of the spinal cord (segments T12-Co3) from MOG-IL6-DIO-Het than MOG-IL6-DIO-ON^Cx3cr1^ mice and were undetectable in MOG-IL6-DIO-KO mice at 27 dpi, even though no differences were significant (Fig 6a, top left). However, *Il6* levels were in line with the disease extent (AUC) (Fig. 6b). In addition, to dissect the putative role of microglial IL-6 in EAE dynamics, we also analyzed the expression of several genes related to different subsets of T cells (*Rorc*, which encodes ROR*yt* protein, and *Foxp3*) and *Il17a* expression. The expression of all these genes was higher in MOG-IL6-DIO-Het mice than MOG-IL6-DIO-KO and MOG-IL6-DIO-ON^Cx3cr1^ mice, but no differences were seen between MOG-IL6-DIO-KO and MOG-IL6-DIO-ON^Cx3cr1^ mice (Fig. 6a).

**Fig. 7:**
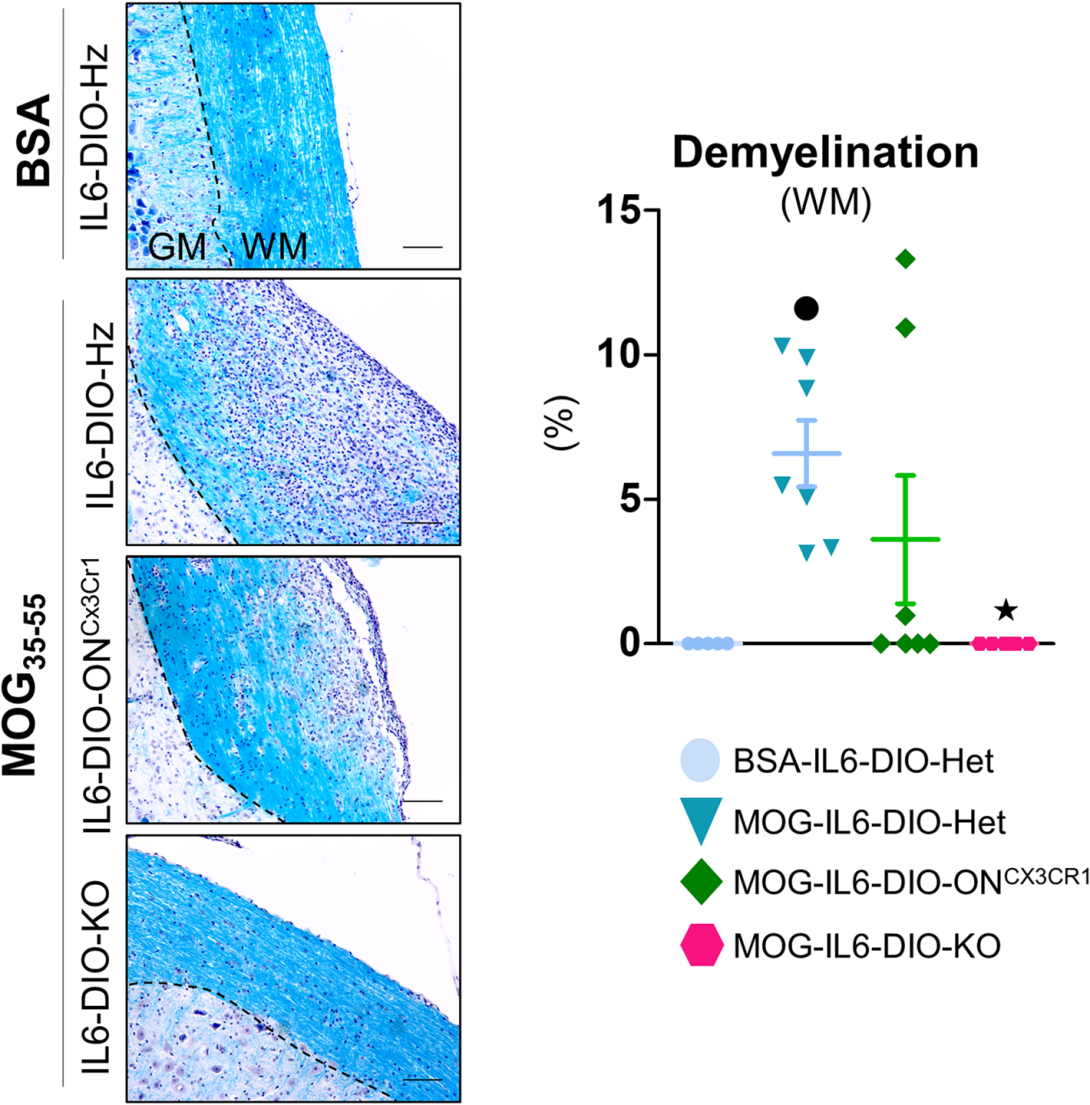
Demyelination was mildly affected by the recovery of microglial IL-6 at 27 dpi. Representative images of LFB/H&E staining and percentage of demyelination in the white matter of the spinal cord from BSA-immunized IL6-DIO-Het and MOG_35-55_-immunized IL6-DIO-Het, IL6-DIO-ON^Cx3cr1^, and IL6-DIO-KO mice. A prominent accumulation of cells was shown in MOG-IL6-DIO-Het mice, and to a lesser extent in MOG-IL6-DIO-ON^Cx3cr1^ mice. The contrast of the representative images was enhanced. The discontinuous line delimits white matter (WM) from gray matter (GM). Scale bar: 100 μm. Results were normalized to the total area and are represented as mean ± SEM; ●*p* ≤ 0.05 vs. BSA-IL6-DIO-Het mice; ★*p* ≤ 0.05 vs. MOG-IL6-DIO-Het mice

In accordance with the clinical signs, the upper part of the spinal cord (segments C1–T11) from MOG-immunized IL6-DIO-Het mice, but not those immunized with BSA, showed demyelination of the white matter. Moreover, a significant effect of IL-6 was observed, since MOG-immunized IL6-DIO-KO mice were practically equal to those of BSA-immunized IL6-DIO-Het mice, while recovery of microglial IL-6 in MOG-IL6-DIO-ON^Cx3cr1^ mice, reverted the demyelination (seemingly partially, but it was not significantly different from MOG-immunized IL6-DIO-Het mice) (Fig. 7).

**Fig. 8:**
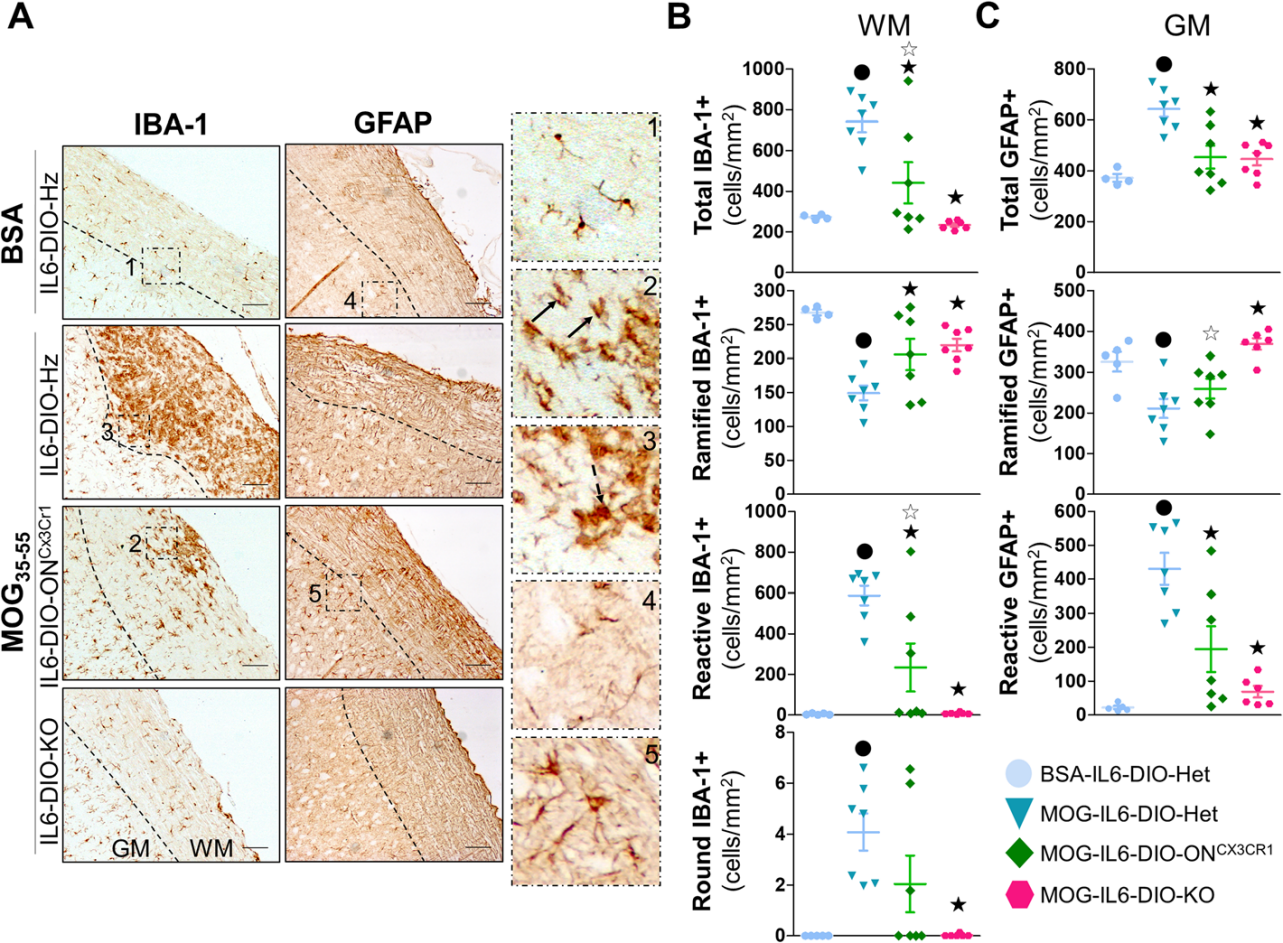
Microglial IL-6 plays a minor role in the regulation of microgliosis and astrogliosis. **a** Representative images of IBA-1 (*left*) and GFAP (*middle*) immunostaining of BSA-immunized IL6-DIO-Het and MOG_35-55_-immunized IL6-DIO-Het, IL6-DIO-ON^Cx3cr1^ and IL6-DIO-KO mice. High magnification (*right*) of (**a**) showing the different morphologies of IBA-1+ and GFAP+ cells: (1) ramified, resting IBA-1+ cells; (2) reactive IBA-1+ cells, with shorter and thicker cell processes (black arrows); (3) round IBA-1+ cells (dashed black arrow); (4) Ramified, resting GFAP+ cells; (5) reactive, hypertrophic GFAP+ cells. The contrast of the representative images was enhanced. The discontinuous line delimits white matter (WM) from gray matter (GM). Scale bar: 100 μm. **b** The total number of IBA-1+ cells and the number of IBA-1+ cells showing a resting phenotype (ramified), partially activated (reactive), and fully activated (round) counted in the white matter of the spinal cord from BSA-immunized WT and MOG_35-55_-immunized IL6-DIO-Het, IL6-DIO-ON^Cx3cr1^, and IL6-DIO-KO mice. **c** The total number of GFAP+ cells and the number of GFAP+ cells showing a resting phenotype (ramified) and activated (reactive) counted in the gray matter of the spinal cord from BSA-immunized WT and MOG_35-55_-immunized IL6-DIO-Het, IL6-DIO-ON^Cx3cr1^, and IL6-DIO-KO mice. All results were relativized per total area and are represented as mean ± SEM; ●*p* ≤ 0.05 vs. BSA-IL6-DIO-Het mice; ★*p* ≤ 0.05 vs. IL6-DIO-Het mice. ☆*p* ≤ 0.05 vs. IL6-DIO-KO mice

MOG_35-55_-immunization also caused an increase of microgliosis (analyzed both by counting IBA-1+ cells and quantitating IBA-1 immunostaining levels) in the white and gray matter of the upper part of the spinal cord (segments C1–T11) from IL6-DIO-Het mice compared with those immunized with BSA. In the white matter, MOG-IL6-DIO-Het showed more microgliosis than MOG-IL6-DIO-KO and MOG-IL6-DIO-ON^Cx3cr1^ mice (Fig. 8a and b, and Additional file 5, left top). Although the IBA-1 immunostaining levels in the white matter of MOG-IL6-DIO-ON^Cx3cr1^ and MOG-IL6-DIO-KO mice were not different, more IBA-1+ cells were counted in MOG-IL6-DIO-ON^Cx3cr1^ than MOG-IL6-DIO-KO mice. In the gray matter, MOG-IL6-DIO-Het showed more IBA-1 immunostaining levels than MOG-IL6-DIO-KO mice, but not compared with MOG-IL6-DIO-ON^Cx3cr1^ mice (Fig. 8a, left, and Additional file 5, left bottom). These results were similar to those for demyelination and suggesting that recovery of microglial IL-6 in MOG-immunized IL6-DIO-ON^Cx3cr1^ mice might partially revert the microgliosis in this spinal cord.

A detailed study about the morphology of IBA-1+ cells (microglia/macrophages) in the white matter was also carried out (Fig. 8a, *right*, and 8b, differentiating three kinds of cells: ramified cells (resting; Fig. 8a, right, panel 1), reactive cells (activated; Fig. 8a, right, panel 2), and round cells (fully activated; Fig. 8a, right, panel 3). The 98.2 ± 0.72 % of IBA-1+ cells from negative control (BSA-injected) IL6-DIO-Het mice displayed a ramified morphology. Similar results were seen in MOG-IL6-DIO-KO since 96.7 ± 0.70% of IBA-1 cells displayed a ramified morphology whereas 3.3 ± 0.7% seemed to take reactive morphology. Among all cell types, the reactive IBA-1+ cells were the largest population (78.9 ± 1.70%) present in MOG-IL6-DIO-Het mice. The IL6-DIO-ON^Cx3cr1^ mice, however, displayed 64.9 ± 14.7% and 34.9 ± 14.5% of ramified and reactive IBA-1+ cells, respectively. A treatment-dependent effect was observed in all three groups of IBA-1 + cells. Regarding MOG-immunized mice, MOG-IL6-DIO-Het mice showed more ramified and reactive IBA-1+ cells compared with MOG-IL6-DIO-KO and IL6-DIO-ON^Cx3cr1^ mice, and IL6-DIO-ON^Cx3cr1^ mice showed more reactive, but not ramified IBA-1+ cells compared with MOG-IL6-DIO-KO mice. Concerning round IBA-1+ cells, a significant increase was appreciated in MOG-IL6-DIO-Het compared with MOG-IL6-DIO-KO mice whereas a trend (*p* = 0.098) was shown in MOG-IL6-DIO-Het as to IL6-DIO-ON^Cx3cr1^ mice. The same trend was observed among MOG-IL6-DIO-KO and IL6-DIO-ON^Cx3cr1^ mice (*p* = 0.098).

In addition, we analyzed the astrogliosis by counting GFAP+ cells and studying GFAP immunostaining levels in the upper part of the spinal cord (segments C1–T11). In particular, no differences between treatments and genotypes were appreciated concerning GFAP immunostaining levels in the white matter, perhaps because of the myelin tracts also seemed to be immunostained, thus increasing background levels (Fig. 8a, middle and Additional file 5, right top). Regarding the gray matter, MOG_35-55_ immunization showed a trend to increase the GFAP immunostaining levels in IL6-DIO-Het compared with those with BSA immunization (*p* = 0.067), being this difference statistically significant when we counted the number of GFAP+ cells. Both GFAP immunostaining levels and the number of GFAP+ cells were different among MOG-IL6-DIO-Het and MOG-IL6-DIO-KO mice (Fig. 8c and Additional file 5, right bottom). Although GFAP immunostaining levels in the gray matter were not statistically significant between MOG-IL6-DIO-Het and MOG-IL6-DIO-ON^Cx3cr1^ mice, we counted a higher number of GFAP+ cells in MOG-IL6-DIO-Het than MOG-IL6-DIO-ON^Cx3cr1^ mice in this matter (Fig. 8c and Additional file 5, right bottom).

We also classified the number of astrocytes in the gray matter of the spinal cord according to their ramified (Fig. 8a, right, panel 4) or reactive (Fig. 8a, right, panel 5) morphology. In IL6-DIO-Het mice immunized with BSA, 93.5 ± 1.15 % of GFAP+ cells displayed a resting ramified morphology, while in those immunized with MOG_35-55_, 33.9 ± 4.76 % had a ramified morphology and 66.1 ± 4.76 % GFAP+ cells seemed to take a star-shaped morphology. In contrast, 85.0 ± 2.93% and 63.2 ± 10.30% of GFAP+ cells displayed a resting morphology in the gray matter of MOG-IL6-DIO-KO and IL6-DIO-ON^Cx3cr1^ mice, respectively. MOG_35-55_-immunization decreased ramified GFAP+ cells, but increased reactive GFAP+ cells in IL6-DIO-Het mice compared with those with BSA-immunization. The number of ramified GFAP+ cells were lower in MOG-IL6-DIO-Het and MOG-IL6-DIO-ON^Cx3cr1^ than MOG-IL6-DIO-KO mice, and a trend to increase the number of ramified GFAP+ cells was also appreciated in MOG-IL6-DIO-ON^Cx3cr1^ compared to MOG-IL6-DIO-Het mice (*p* = 0.081). Genotypic differences were also observed regarding the number of GFAP+ cells with a reactive morphology, which was higher in MOG-IL6-DIO-Het than MOG-IL6-DIO-KO and MOG-IL6-DIO-ON^Cx3cr1^ mice. The recovery of microglial IL-6 in MOG-IL6-DIO-ON^Cx3cr1^ mice tended to increase the number of reactive GFAP+ cells in comparison with MOG-IL6-DIO-KO mice (*p* = 0.063; Fig. 8b, right).

## Discussion

Traditional methods for creating reversible KO mice are based on strategies that require numerous crossings or the continuous administration of drugs. The main methods are (1) cassette stop/trap flanked by loxP sites [33–35], (2) Tet-off and tet-on [36–38], and (3) LOFT: LoxP-flippase (FLP) recognition target (FRT) Trap [39].

The stop-cassette strategy is based on creating a knockout by introducing transcription termination elements that prevent the complete production of the target protein. Reversibility is achieved by driving Cre expression with tissue-specific promoters fused with the estrogen receptor (ER) paired with tamoxifen injection for temporal and spatial specificity. Alternatively, injection of a Cre-expressing virus can rescue in a similarly time- and spatial-specific manner. However, stop cassettes can be leaky and trace expression must be assessed empirically.

Tet-off and Tet-on use Escherichia coli’s tetO operon to induce expression of the adjacent gene. In the “off” system, the mouse becomes a KO upon (and only during) administration of tetracycline (or, in practice, its derivative doxycycline), which then binds to a tetracycline-controlled transactivator (tTA) that has been engineered to be constitutively expressed, and prevents the transcription of the gene downstream of tetO. In the “on” system, the transactivator is modified to have the reverse phenotype (rtTA), i.e., it only binds to tetO in the presence of doxycycline. In this case, the animal is initially a KO, and it will only produce the target protein upon drug administration. As downsides, both strategies depend on continuous administration of the drug to maintain the animal’s genotype; the use of a stop cassette with loxP sites in order to obtain tissue specificity has the problem mentioned above; and furthermore, they require extensive breeding and animals that are transgenic for multiple genes.

The LOFT system uses two modified alleles for the gene of interest, one of them floxed and the other with an frt-flanked gene-trap cassette (that also has Neo and GFP) inserted in an intron. The latter will produce by default an inactive fusion protein with the N-terminal domain of the target protein and Neo. When Cre is expressed, the animal will become a KO. If there is additionally flippase activity, the phenotype will be reversed. This method requires a considerable number of crossings to incorporate the two alleles and recombinases making it labor- and cost-intensive. In addition, given that the allele with the gene-trap is null by default, the gene must be haplosufficient.

To overcome these pitfalls, we have generated a conditional and reversible KO mouse using the widely used in opto- and chemogenetics double-inverted, open-reading-frame paradigm [4, 29–31]. This technique consists of flanking exons of interest by pairs of incompatible loxP sites such that Cre recombinase flips the exons and then locks them into the flipped orientation [4, 29–31]. Exon 2 of the *Il6* gene was inverted in IL6-DIO-KO mice causing a depletion of IL-6 protein (undetectable after inflammation challenges such as LPS and EAE), which is equivalent to results with the total IL-6 KO mice [12, 40]. In our EAE paradigm, almost all IL6-DIO-KO mice were resistant to MOG_35-55_-inducible EAE, except for one, which presented EAE-related symptoms even without showing serum IL-6 levels, as previously observed with other IL-6 KO models [11, 12, 41].

The EAE-related symptoms of IL6-DIO-KO mice were similar to those of WT mice immunized with MOG_35-55_ peptide and administered intraperitoneally with anti-IL-6 receptor monoclonal antibody (clone MR16-1) at the same day of EAE induction, but different to those of WT mice immunized with MOG_35-55_ peptide and treated with clone MR16-1 after disease onset [42]. In addition, WT mice immunized with MOG_35-55_ peptide and orally treated during ongoing EAE with tocilizumab, a humanized monoclonal antibody that inhibits IL-6R-mediated signaling, showed a drastic reduction of EAE-related symptoms [43]. It is therefore clear that IL-6 signaling is involved in the pathogenesis of EAE. However, given the functional complexity of IL-6, the priority of many groups during the last decades has been to study the multiple cellular sources of IL-6 to better understand the contribution of each one to the EAE pathogenesis. Until now, the roles of IL-6 in a specific context have been described through the use of mice lacking IL-6 throughout the body or in specific cells (total and conditional IL-6 KO mice, respectively). Nevertheless, the interpretation of the results using the latter model is difficult because the data could be the result of a compensatory response from IL-6 of other cells. With our new conditional reversible IL-6 KO mouse, we now have a tool that allows for the reactivation of *Il6* expression in cells expressing Cre recombinase. Thus, we were able to recover microglial IL-6 in the reversible total IL-6 KO mouse through breeding to *Cx3cr1*-CreER mice and subsequent TAM administration. Our results suggest that recovery of microglial IL-6 gives rise to a mild version of EAE, albeit with a limited role in the regulation of the inflammatory cascade. In conclusion, we have verified that the new reversible total IL-6 KO is an excellent tool to study the role of specific cellular sources of IL-6 within a recovery-of-function paradigm.

Although EAE is considered a peripheral disease, microglia, besides CD4+ cells, have been described as responsible for EAE initiation [23, 44]. In fact, in MOG_35-55_-immunized C57BL/6 mice, CD4+ T cells infiltrated the brain before mice developed EAE-related symptoms, coinciding with activation of CD11b+ microglia and production of IL-1β, TNF-α, and IL-6 [45]. Furthermore, we have recently observed that microglia are one of the main brain sources of IL-6 during EAE pathogenesis and microglial *Il6*-deficient females seemed to be less affected by MOG_35-55_ immunization than their controls [19]. In this paper, we have focused on studying the effect of selective recovery of microglial IL-6 expression by crossing IL6-DIO-KO with Cx3cr1-CreER mice to show the applicability of this new system and to gain insight into the putative role(s) of microglial IL-6 in the EAE disease.

In Cx3Cr1CreER mice, Cre is driven by the *Cx3cr1* promoter, which is broadly expressed in the mononuclear phagocytic cells and it is only expressed in microglia in the adult brain [7, 46, 47]. Furthermore, Cre recombinase protein of these mice is fused to the estrogen ligand-binding domain (CreER), thereby requiring TAM for its activation [6, 7]. Then, IL6-DIO-ON^Cx3cr1^ mice should revert *Il6* expression in Cx3cr1-expressing cells, including macrophages and microglia, after TAM treatment. Since IL-6 protein levels in serum are practically undetected in basal conditions, mice were also injected with LPS following TAM treatment to increase IL-6 production. The recovery of IL-6 occurred in IL6-DIO-ON^Cx3cr1^ mice administrated with TAM for 11 days and not for 5 days. However, IL-6 production was lower in LPS-IL6-DIO-ON^Cx3cr1^ than LPS-IL6-DIO-Het mice, probably due to the fact that only CX3CR1+ cells in IL6-DIO-ON^Cx3cr1^ mice would be responsible for IL-6 production following LPS administration while all cells would produce IL-6 protein in IL6-DIO-Het mice (these mice have one WT allele and one DIO allele; in addition, TAM administration would cause the Cre activation and subsequent *Il6* reversion of the DIO allele in CX3CR1+ cells in these mice). We did not attempt longer TAM administrations given that our current dose is already at the higher end of the reported spectrum [48, 49] and the drug is a known immunosuppressant which could interfere with EAE evaluation [50].

The longevity and limited self-renewal are the main features of microglia which distinguishes them from other mononuclear phagocytic cells [51, 52]. In fact, Goldmann and colleagues observed that inducible Cx3cr1CreER:R26-YFP mice, obtained by crossing Cx3Cr1CreER mice with R26-YFP mice, displayed YFP-labeled microglia at all times after TAM treatment whereas other mononuclear phagocytic cells lost the YFP-label for the first 4 weeks after treatment [6]. We also observed similar results using our inducible microglial IL-6 KO mice, obtained by crossing Cx3Cr1CreER mice with *Il6*^*l*ox/lox^ mice, since microglia of these mice, but not other brain cells or liver (assuming this tissue as representative of macrophages/monocytes), kept the genomic modification 4 weeks following TAM administration [26]. Therefore, we also performed the validation of IL6-DIO-ON^Cx3cr1^ mice at the same time as above mentioned. As expected, IL6-DIO-ON^Cx3cr1^ mice kept the reversion of the DIO allele in microglia, but not in other brain cells or liver, 4 weeks after being treated with TAM for 11 days. These data confirm that the contribution of mononuclear phagocytic cells to results obtained with IL6-DIO-ON^Cx3cr1^ mice from 4 weeks following TAM-treatment will be limited.

Reactivation of microglial *Il6* expression in MOG_35-55_-immunized IL6-DIO-ON^Cx3cr1^ females developed some of the prototypical paralyzing symptoms showing that despite the fact that EAE is considered essentially a peripheral disease, microglial *Il6* was sufficient to trigger a mild version of EAE-related symptoms. Results are in line with females lacking IL-6 in microglia, which tended to reduce EAE score during the acute phase of the disease in comparison with their controls [19]. Clinical signs of IL6-DIO-ON^Cx3cr1^ mice were lower than those of IL6-DIO-Het animals, reinforcing the idea that IL-6 derived from other cellular sources is also involved in the EAE-related symptomatology. Of note, scores (AUC) within the IL6-DIO-ON^Cx3cr1^ group have a bimodal distribution, which also does not fully correlate with *Il6* expression. This could be due to only partial recovery of microglial *Il6* or to this source not being as relevant to disease progression and just indicating a stochastic process. Indeed, T-cell- and astrocyte-derived *Il6-*deficient mice showed ameliorated symptoms compared with their controls after being immunized with MOG_35-55_ [17–19]. However, deficiency of dendritic cell-derived *Il6* caused that naïve T cells could not differentiate into Th17 cells blocking EAE score in mice [17]. In contrast, *Il6* deficiency in other cells caused no (in neurons), contradictory (in B cells), or aggravated (macrophage) effects in the regulation of EAE-related symptoms [16, 17, 19].

The expression of mRNA encoding *Il6* was higher in the lower part of the spinal cord (segments T12-Co3) from MOG-IL6-DIO-Het than MOG-IL6-DIO-ON^Cx3cr1^ mice and it was absent in MOG-IL6-DIO-KO mice at 27 dpi, but high variability precluded any statistically significant effect between genotypes. Moreover, we cannot rule out that differences would exist at other time points. EAE is a complex disease in which IL-6 undoubtedly plays an important role since it suppresses the differentiation of TGF-β-induced Foxp3+ Treg, and with TGF-β promotes the differentiation of pathogenic TH17 cells from naive T cells [15]. In this paper, IL6-DIO-KO mice showed downregulation of *Rorc* expression, the crucial transcription factor of Th17, suggesting a reduction of Th17 differentiation, and a similar trend regarding *Il17* mRNA levels in the spinal cord compared to IL6-DIO-Het. Indeed, less percentage of Th17 cells infiltrating the CNS was previously reported using MOG_35-55_-immunized IL-6 KO mice [15]. Surprisingly, the recovery of microglial IL-6 did not modify *Il17* expression in comparison with the lack of total IL-6 in mice at 27 dpi, as it was observed in IL-17 immunostaining levels in microglial *Il6* deficient mice at 27–29 dpi [19]. Besides, the expression of mRNA encoding *Foxp3,* the master transcription factor of Treg, was also downregulated by lack of total IL-6, in agreement with IL-6 KO mice, whose cerebellum showed less percentage of Foxp3 cells per infiltrate than those of MOG_35–55_-immunized WT mice [12]. Interestingly, another work reported that IL-6 KO mice (crossed previously with the Foxp3gfp.KI mice to monitor Treg cells) showed more Foxp3+ Treg cells in the draining lymph nodes than their controls [53], which suggests the necessity of further studies to determine these discrepancies. *Foxp3* and *Rorc* expression levels were practically equal among MOG-IL6-DIO-ON^Cx3cr1^ and MOG-IL6-DIO-KO mice at 27 dpi, but distinct to MOG-IL6-DIO-Het, suggesting that other cellular sources of IL6, and not microglia, might regulate these transcription factors at this time point. IL-6 has also been described as a regulatory protein of some inflammation-related genes at 15-20 dpi [12, 54]. In particular, microglial *Il6* deficiency caused changes in *Gfap* and *Cd206* (alternatively activated macrophages/microglia (M2) phenotypic marker) expression in the lower part of the spinal cord (segments T12-Co3) from mice at 15 dpi [19]. Future studies are needed to determine what occurs regarding inflammation-related markers in the MOG-IL6-DIO-ON^Cx3cr1^ mice at peak disease.

The histological analysis of the upper part of the spinal cord (segments C1–T11) from MOG-IL6-DIO-KO mice revealed a decrease of demyelination and limited invasion of CD3+ in the white matter, as previously observed using other IL-6 KO models immunized with MOG_35-55_ [11, 12]_,_ and highlighting once again a critical role of IL-6 in the prototypical autoimmune disorder. Interestingly, the recovery of microglial IL-6 in MOG-IL6-DIO-ON^Cx3cr1^ females seemed to revert partially the demyelination, in agreement with results obtained with microglial *Il6*-deficient females at 29 dpi, which showed a trend to decrease the demyelination [19], pointing to in both cases that IL-6 derived from microglia might regulate the demyelination. As stated, another key pathological feature of EAE is the inflammatory response of astrocytes and microglia/macrophages, therefore we quantified the GFAP and IBA-1 immunostaining levels and classify IBA-1 and GFAP+ cells according to their morphology. In general, the lack of IL-6 reduced the microgliosis and astrogliosis in the spinal cord of mice, displaying most of IBA-1+ and GFAP+ cells a resting morphology, in line with IL-6 KO [12]. The recovery of microglial IL-6, in agreement with the mild clinical signs of MOG-IL6-DIO-ON^Cx3cr1^ mice, increased the number of reactive IBA-1+ cells in the white matter and a similar trend was observed regarding IBA-1 immunostaining levels in the gray matter. Somewhat surprisingly, considering the relevance of IL-6 in inflammatory processes of EAE, is that the recovery of microglial IL-6 in MOG-IL6-DIO-ON^Cx3cr1^ mice did not modify the total number of GFAP+ cells in the gray matter, although it reduced the ramified GFAP+ cells and tended to increase the reactive GFAP+ cells. Taking into account the results obtained with microglial *Il6*-deficient females at 29 dpi, in which deficiency of microglial *Il6* tended to reduce IBA-1 immunostaining levels in the white matter, but it did not play a relevant role in the regulation and GFAP immunostaining levels [19], it seems that microglial *Il6* may regulate the microgliosis, but it would have a limited function in the regulation of astrogliosis during the inflammatory response to EAE.

## Conclusions

In summary, we have presented and validated a novel reversible total IL-6 KO mouse, whose *Il6* expression was recovered specifically in microglia. The recovery of microglial *Il6* was sufficient to develop a mild version of EAE-related symptoms in females, although it seemed to have a minor role in the regulation of the inflammatory cascade. Furthermore, the reversion of *Il6* expression in different cells using this IL6-DIO-KO mouse model, combined with cell-specific Cre transgenic lines, would be key to elucidate the specific functions of IL-6 from each cellular source and study cell-specific IL-6 targeting strategies in EAE and a wide range of autoimmune and neuroinflammatory diseases. Indeed, our group is currently analyzing the role of IL-6 in a mouse model of Alzheimer’s disease and in a mouse model of progressive encephalopathy resembling Leigh syndrome using this novel strategy. Furthermore, the IL6-DIO-KO mouse model will be available for the scientific community and will be distributed worldwide following material transfer agreements with Universitat Autònoma de Barcelona. Finally, we also encourage other investigators to take up this reversible and conditional knock-out strategy to carry out rescue experiments in other genetic models.

## Supplementary information

**Additional file 1. **Probe sequence. Primers *Fw*:5’-CAGCATCTCATCTGAGTTCCG-3’ and *Rv*:5’-CTCACTGTTCACAAAGCACAGG-3’ were used to design a unique probe of 621 bp.

**Additional file 2.** DNA sequencing of the modified *Il6* gene of the IL6-DIO-KO mice. The resulting sequence had 1361 bp and the localization, size and color code of the genetic structures (exons and protein binding zones) detected in this sequence are explained in the figure legend. The loxP and lox2272 cassettes (purple and green, respectively) are in the opposite orientation, then sequence between them (including the exon 2) will be reverted after Cre action.

**Additional file 3.** Kaplan-Meier analysis in the EAE experiment with IL6-DIO-KO and WT mice. Mice were assigned status “sick” on the first day their score was ≥1 and the proportion of sick mice was represented for each day. Mice that never showed scores above the threshold were censored. The time-course of disease for IL6-DIO-KO mice is significantly delayed, with only one mouse fulfilling the disease criterion (no median). Table shows number of mice, sick mice, median day and 95% confidence interval. NA (not applicable) indicates impossibility to calculate value.

**Additional file 4.** Kaplan-Meier analysis in the EAE experiment with IL6-DIO-ON^Cx3cr1^, IL6-DIO-KO and IL6-DIO-Het mice. Mice were assigned status “sick” on the first day their score was ≥1 and the proportion of sick mice was represented for each day. The time-course of disease for IL6-DIO-ON^Cx3cr1^ and IL6-DIO-KO mice is significantly delayed. IL6-DIO-KO again showed almost complete resistance to EAE, with only one mouse actually developing the disease (no median). Tables show number of mice, sick mice, median day and 95% confidence interval; as well as *post-hoc* pair-wise comparisons. NA (not applicable) indicates impossibility to calculate value.

**Additional file 5.** Quantification of IBA-1 and GFAP immunostaining levels shown in Fig. 8. Quantification was carried out in both white and gray matter of BSA-immunized IL6-DIO-Het and MOG_35-55_-immunized IL6-DIO-Het, IL6-DIO-ON^Cx3cr1^ and IL6-DIO-KO mice. All results were relativized per total area and are represented as mean ± SEM; ●*p* ≤ 0.05 vs. BSA-IL6-DIO-Het mice; ★*p* ≤ 0.05 vs. IL6-DIO-Het mice.

## Data Availability

The datasets used and/or analysed during the current study are available from the corresponding author on reasonable request.

## Abbreviations

AUC: Area under the curve
BSA: Bovine serum albumin
CD11b: Cluster of differentiation molecule 11b
CNS: Central nervous system
DIO: Double-inverted open reading frame
EAE: Experimental autoimmune encephalomyelitis
GFAP: Glial fibrillary acidic protein
GM: Gray matter
IBA-1: Ionized calcium-binding adaptor molecule-1
IL-6: Interleukin-6
LFB: Luxol Fast Blue
LOFT: LoxP-flippase (FLP) recognition target (FRT) Trap
LPS: Lipopolysaccharide
MOG_35-55_: Myelin oligodendrocyte glycoprotein 35-55 peptide
MS: Multiple sclerosis
TAM: Tamoxifen
WM: White matter
YFP: Yellow fluorescent protein
