# Supplementary figures and images for: A new mouse model to study restoration of interleukin-6 (IL-6) expression in a Cre-dependent manner: microglial IL-6 regulation of experimental autoimmune encephalomyelitis
Source: J Neuroinflammation. 2020 Oct 15;17:304. doi: 10.1186/s12974-020-01969-0 (PMC7565836; doi:10.1186/s12974-020-01969-0)

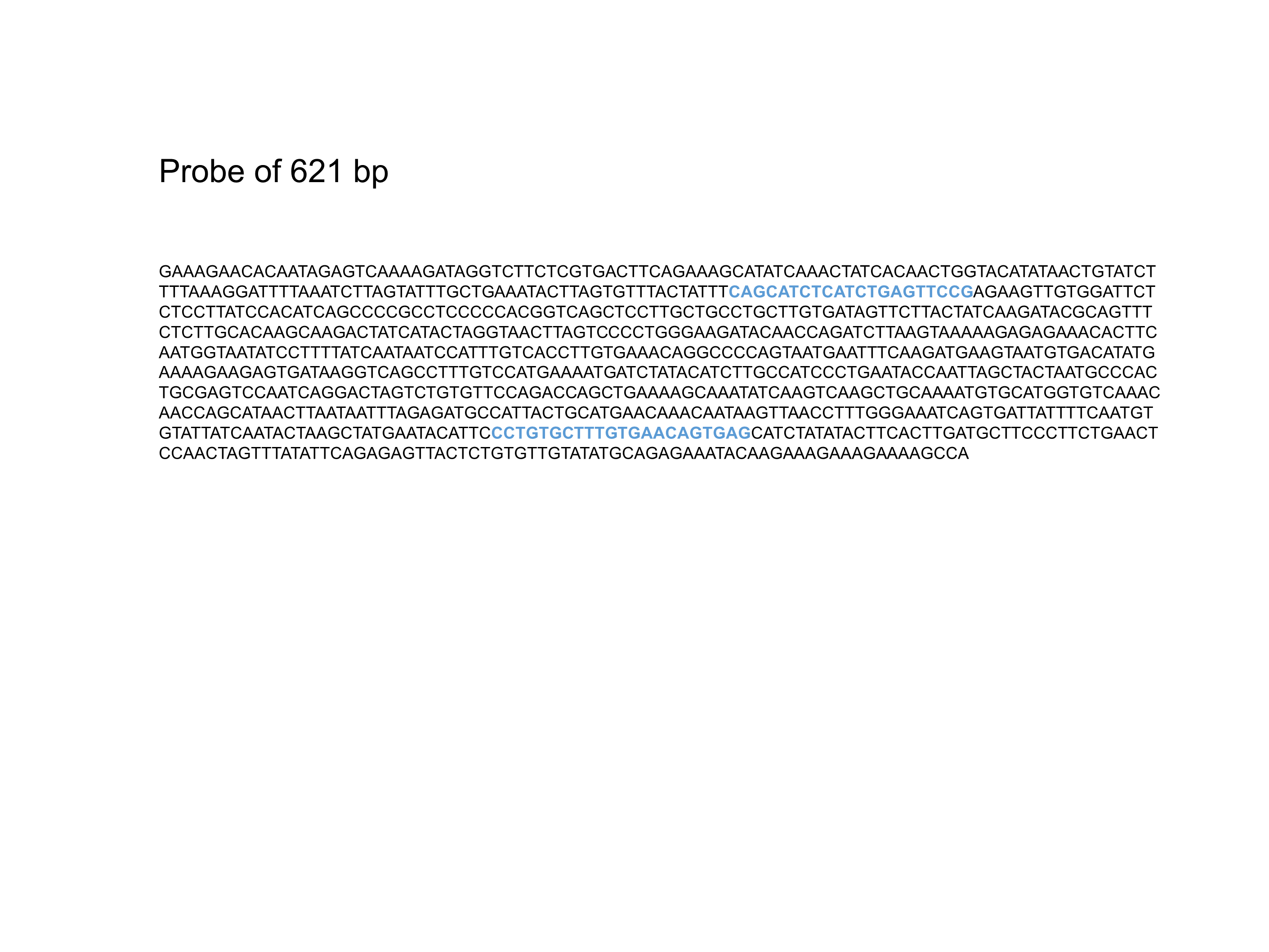

Supplement: Supplementary file 1 — Additional file 1. Probe sequence. Primers Fw:5’-CAGCATCTCATCTGAGTTCCG-3’ and Rv:5’-CTCACTGTTCACAAAGCACAGG-3’ were used to design a unique probe of 621 bp. [file 12974_2020_1969_MOESM1_ESM.tif]

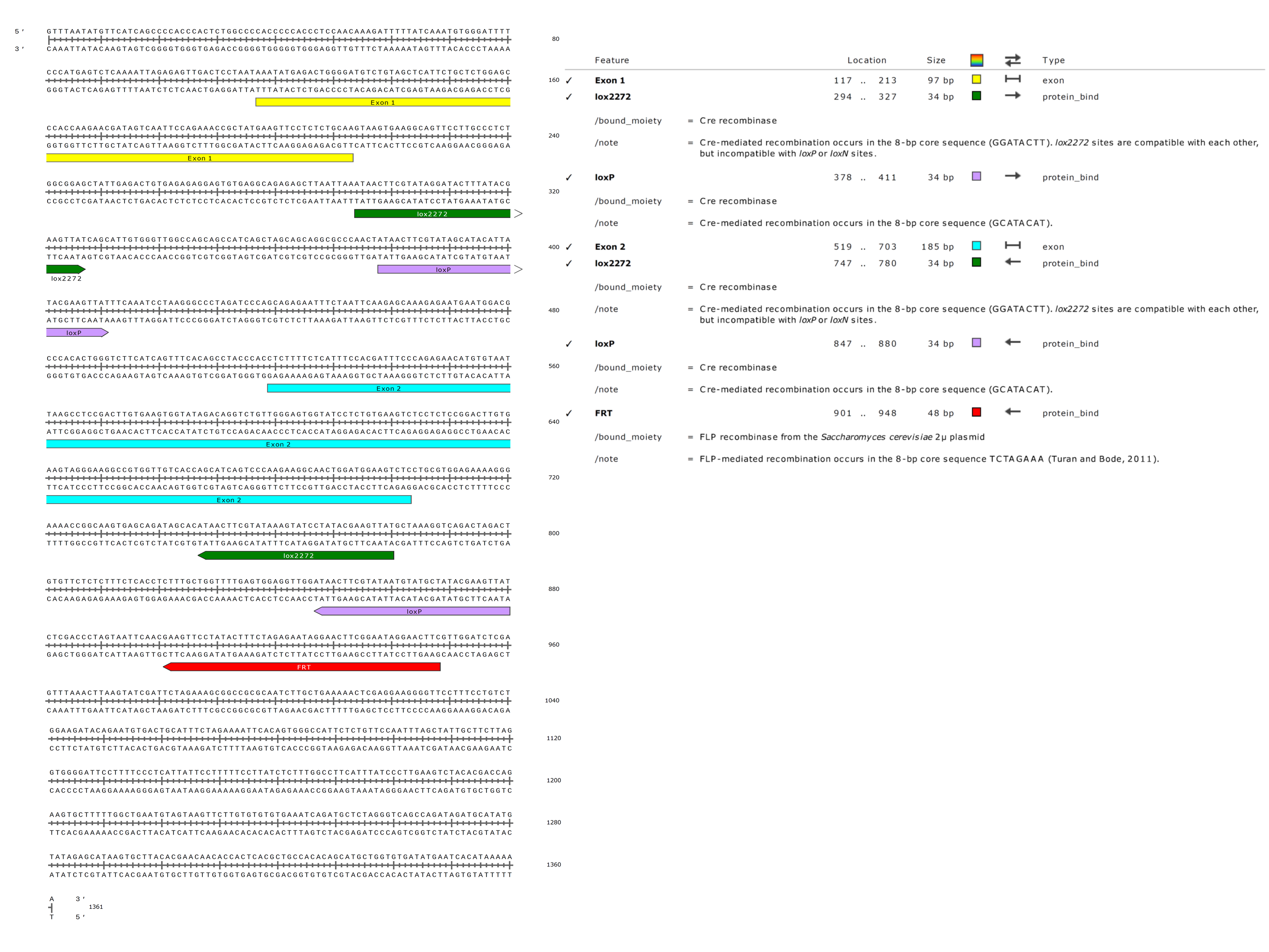

Supplement: Supplementary file 2 — Additional file 2. DNA sequencing of the modified Il6 gene of the IL6-DIO-KO mice. The resulting sequence had 1361 bp and the localization, size and color code of the genetic structures (exons and protein binding zones) detected in this sequence are explained in the figure legend. The loxP and lox2272 cassettes (purple and green, respectively) are in the opposite orientation, then sequence between them (including the exon 2) will be reverted after Cre action. [file 12974_2020_1969_MOESM2_ESM.tif]

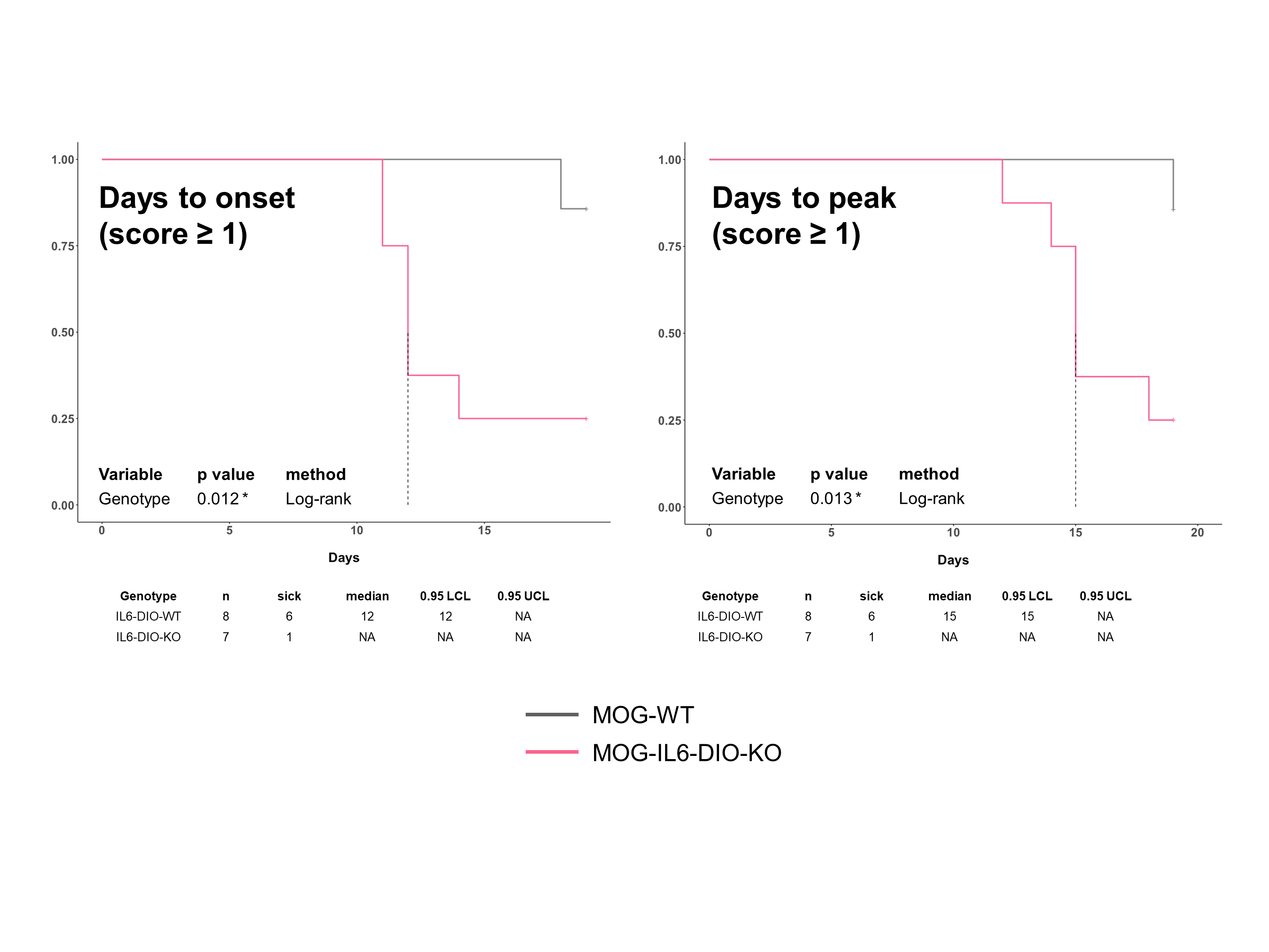

Supplement: Supplementary file 3 — Additional file 3. Kaplan-Meier analysis in the EAE experiment with IL6-DIO-KO and WT mice. Mice were assigned status “sick” on the first day their score was ≥1 and the proportion of sick mice was represented for each day. Mice that never showed scores above the threshold were censored. The time-course of disease for IL6-DIO-KO mice is significantly delayed, with only one mouse fulfilling the disease criterion (no median). Table shows number of mice, sick mice, median day and 95% confidence interval. NA (not applicable) indicates impossibility to calculate value. [file 12974_2020_1969_MOESM3_ESM.tif]

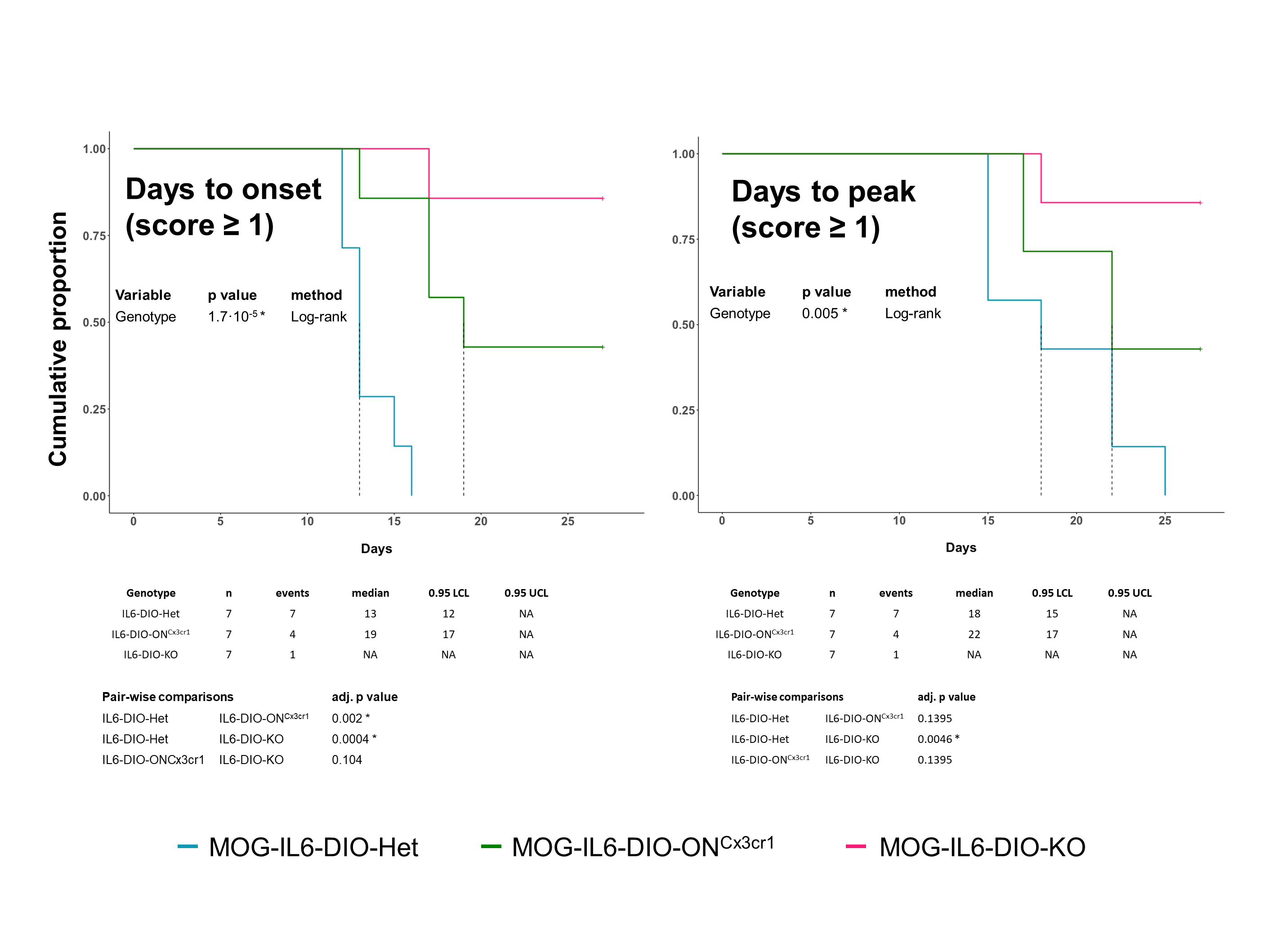

Supplement: Supplementary file 4 — Additional file 4. Kaplan-Meier analysis in the EAE experiment with IL6-DIO-ONCx3cr1, IL6-DIO-KO and IL6-DIO-Het mice. Mice were assigned status “sick” on the first day their score was ≥1 and the proportion of sick mice was represented for each day. The time-course of disease for IL6-DIO-ONCx3cr1 and IL6-DIO-KO mice is significantly delayed. IL6-DIO-KO again showed almost complete resistance to EAE, with only one mouse actually developing the disease (no median). Tables show number of mice, sick mice, median day and 95% confidence interval; as well as post-hoc pair-wise comparisons. NA (not applicable) indicates impossibility to calculate value. [file 12974_2020_1969_MOESM4_ESM.tif]

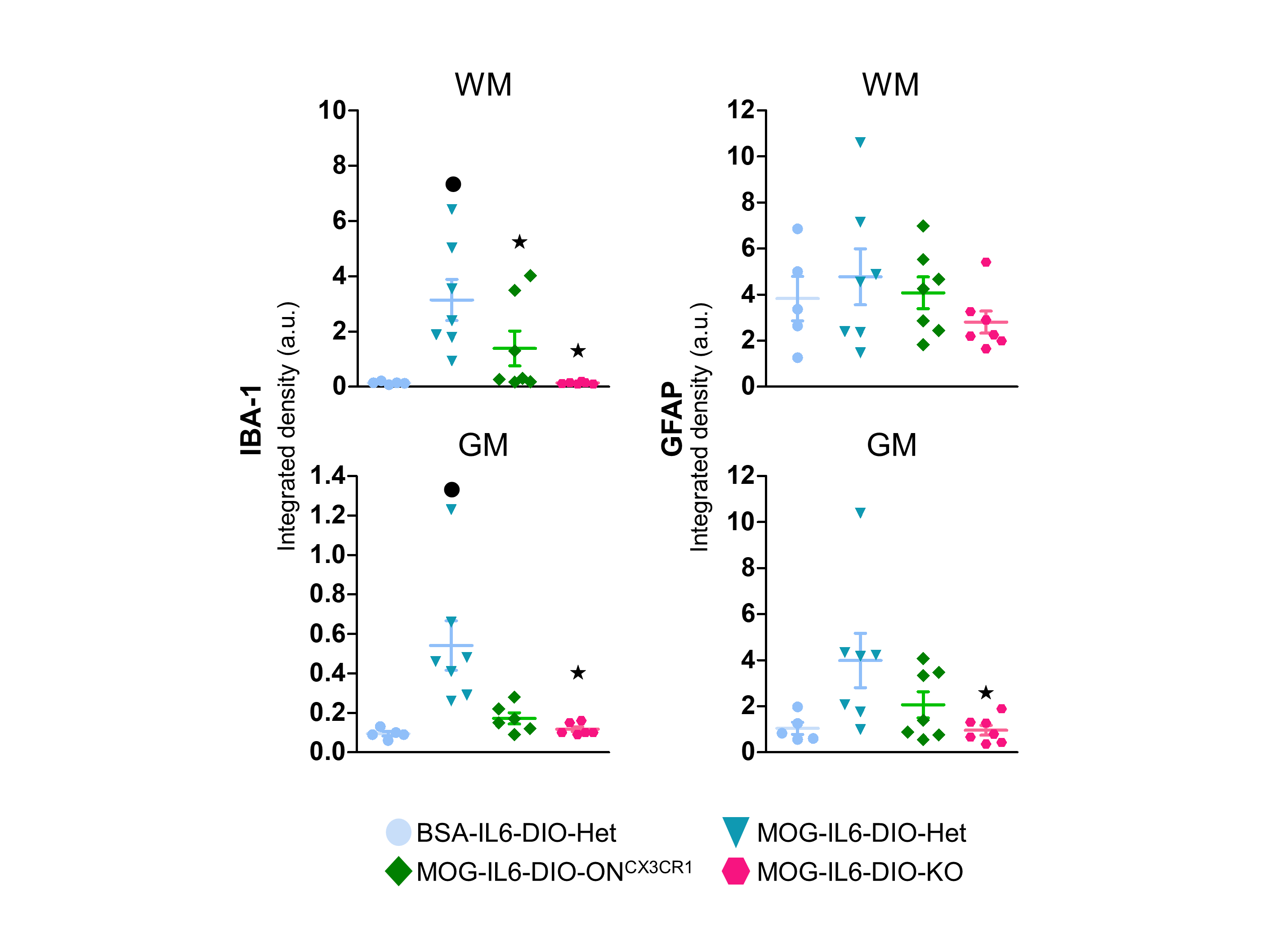

Supplement: Supplementary file 5 — Additional file 5. Quantification of IBA-1 and GFAP immunostaining levels shown in Fig. 8. Quantification was carried out in both white and gray matter of BSA-immunized IL6-DIO-Het and MOG35-55-immunized IL6-DIO-Het, IL6-DIO-ONCx3cr1 and IL6-DIO-KO mice. All results were relativized per total area and are represented as mean ± SEM; ●p ≤ 0.05 vs. BSA-IL6-DIO-Het mice; ★p ≤ 0.05 vs. IL6-DIO-Het mice. [file 12974_2020_1969_MOESM5_ESM.tif]
